# General method for the synthesis of enaminones via photocatalysis

**DOI:** 10.3762/bjoc.21.116

**Published:** 2025-07-29

**Authors:** Paula Pérez-Ramos, Raquel G Soengas, Humberto Rodríguez-Solla

**Affiliations:** 1 Department of Organic and Inorganic Chemistry, and Instituto Universitario de Química Organometálica Enrique Moles, University of Oviedo, Julián Clavería 8, 33006, Oviedo, Spain

**Keywords:** chromones, dehalogenation, enaminones, nickel, photocatalysis

## Abstract

Enaminones are key intermediates in the synthesis of several derivatives with important applications in medicinal chemistry. Furthermore, many marketed drugs feature the enaminone structural moiety. In this context, we have developed a photoredox and nickel catalytic system to rapidly forge the enaminone scaffold from 3-bromochromones via a Michael reaction of an amine with an electron-deficient alkene moiety and subsequent photocatalyzed debromination. With this dual catalytic system, a range of structurally diverse enaminone derivatives have been achieved in good yields with total *trans* selectivity. Mechanistic studies indicate that the key to the success of this process is the formation of an unexplored ternary Ni-complex with 3-bromochromone and a pyridinium salt, which is crucial for the effective activation of the α,β-unsaturated system towards the nucleophilic addition.

## Introduction

**Figure 1:**
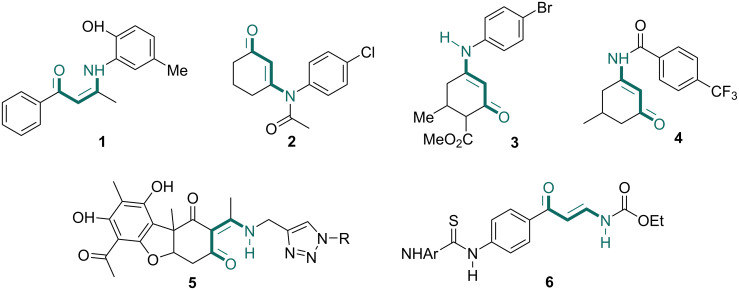
Examples of compounds with medicinal effects containing an enaminone structural moiety.

Enaminones are relevant intermediates in organic chemistry and the pharmaceutical industry [1–6]. These enamines have a carbonyl group conjugated to a carbon–carbon double bond, owing its great versatility in organic synthesis to its ability to act as both electrophiles and nucleophiles [7]. This makes enaminones very reactive, providing an excellent scaffold for organic synthesis. Thus, enaminones are valuable building blocks in the preparation of several carbocyclic [8–11], heterocyclic [12–18] as well as acyclic compounds [19–23]. Furthermore, the enaminone structural moiety represents the key framework of many drug classes, including antibiotic (**1**) [24], anti-inflammatory (**2**) [25], antinociceptive (**3**) [26], anticonvulsant (**4**) [27], antitubercular (**5**) [28], and antitumor (**6**) [29] agents (Figure 1).

In view of the important biological roles of enaminones and their relevance as synthetic intermediates, it is not surprising that there has been a continuous focus on developing general, straightforward, and efficient strategies for their synthesis. Enaminones are usually accessed by condensation of 1,3-dicarbonyl compounds with amines [30]. While this approach is simple and straightforward, it often leads to a mixture of constitutional isomers in which the two different α-positions of the ketone have been functionalized. Therefore, the development of novel methods for the synthesis of enaminones has attracted much attention over the past decades.

**Scheme 1:**
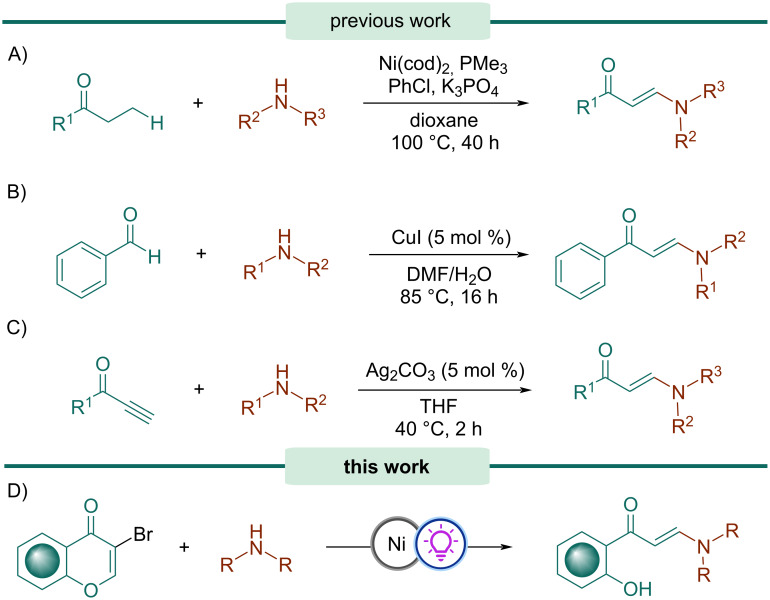
Synthesis of enaminones.

Kuwano’s group described the synthesis of enaminones from ethyl ketones via a nickel-catalyzed selective β-amination (Scheme 1A) [31]. The preparation of enaminones can also be achieved by the reaction of aldehydes and calcium carbide in the presence of amines and CuI as catalyst, as reported by Zhang and co-workers (Scheme 1B) [32]. On the other hand, Li et al. disclosed a silver-catalyzed amination of propargyl alcohols to afford enaminones (Scheme 1C) [33].

Although these new methods provide a wide variety of enaminones, there are limitations such as expensive and unavailable reagents, long reaction times and drastic reaction conditions. Furthermore, the increasing emphasis on economic and environmental factors has highlighted the limitations of traditional methods for enaminone synthesis to align with the modern understanding of organic chemistry.

With the increasing concern on the environmental impact of organic synthesis, photocatalysis emerged as a powerful synthetic tool in organic chemistry, offering new ways to deliver diverse organic products via mild, easy to handle, and environmentally benign operations [34–36]. Thus, the use of visible light as an energy source provides more efficient chemical transformations and minimize the use of harmful reagents, the generation of waste and the consumption of energy, fulfilling several principles of Green Chemistry and promoting greener opportunities for organic synthesis [37–38]. In this context, the reactivity of enaminones under visible-light-mediated reaction conditions has attracted significant attention [39]. However, it is rather surprising that a photocatalytic approach for the synthesis of enaminones has yet to be explored.

Herein, we report the first light-mediated reaction for the synthesis of enaminones from 3-bromochromones (Scheme 1D). Initially, a Ni(II)-catalyzed hydroamination protocol affords the intermediate 2-amino-3-bromochromanones, which upon photocatalytic dehalogenation and subsequent opening of the heterocyclic ring provide the corresponding enaminones. This transformation is simple, straightforward, and proceeds under mild conditions.

## Results and Discussion

The initial challenge in achieving the desired reactivity was the activation of the unsaturated system towards the nucleophilic addition of the amine. The most common strategy to increase the reactivity of unsaturated esters towards an aza-Michael addition is the use of transition metal complexes as catalysts/promoters [40–42]. Considering this background, we reasoned that Ni(II) could be a suitable catalyst for the amination of unsaturated systems.

Initial investigations were carried out by using 3-bromochromone (**7a**) and morpholine (**8a**) as model substrates in the presence of Ni(II) salt (5 mol %) and ligand (5 mol %), a pyridinium salt (1 equiv) and a photocatalyst (1 mol %) under 427 nm blue LEDs. After carefully screening of the reaction parameters (Table 1 and Tables S1–S7, Supporting Information File 1), we found that a combination of 1-[1-(*tert*-butoxycarbonyl)piperidin-4-yl]-2,4,6-triphenylpyridin-1-ium (**PS1**), NiBr_2_·diglyme, 4,4'-dimethoxy-2,2'-bipyridine (dmbpy), and 10-(3,5-dimethoxyphenyl)-9-mesityl-1,3,6,8-tetramethoxyacridin-10-ium tetrafluoroborate (**PC1**) in *N*,*N*-dimethylformamide (DMF) at 20 °C afforded the best results, giving the desired product **9a** in 70% isolated yield (Table 1, entry 1). Changing NiBr_2_·diglyme to other nickel salts, such as Ni(OTf)_2_ and NiCl_2_·diglyme led to lower yields (Table 1, entries 2 and 3). Similarly, changing the ligand for dtbbpy or phenantroline also resulted in a decrease in the efficiency of the process (Table 1, entries 4 and 5). The pyridinium salt has also a significant effect on reactivity; thus, when 1-benzyl-2,4,6-triphenylpyridin-1-ium (**PS2**) was used, enaminone **9a** was isolated in only 34% yield (Table 1, entry 6). Replacing DMF by DME, DMSO or acetone diminished the product yields (Table 1, entries 7–9). The reactivity of acridinium **PC1** was superior to that of other photocatalysts, including 4-CzlPN (**PC2**) and [Ir(dF(CF_3_)ppy)_2_(dtbbpy)]PF_6_ (**PC3**) (Table 1, entries 10 and 11).

**Table 1:** Optimization of reaction conditions.

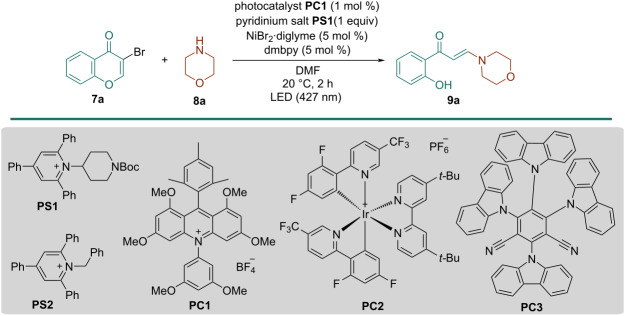		

Entry	Deviation from the standard conditions^a^	Yield (%)^b^

1	none	70
2	Ni(OTf)_2_ instead NiBr_2_·diglyme	62
3	NiCl_2_·diglyme instead NiBr_2_·diglyme	49
4	dtbbpy instead dmbpy	66
5	phenantroline instead dmbpy	49
6	**PS2** instead **PS1**	34
7	acetone instead DMF	51
8	DMSO instead DMF	55
9	DME instead DMF	57
10	**PC2** instead **PC1**	46
11	**PC3** instead **PC1**	57
12	40 °C instead 20 °C	traces
13	16 h instead 2 h	18
14	no **PS**	33
15	no Ni salt and ligand	30
16	no **PC**	n.r.^c^
17	no light	n.r.

^a^3-Bromochromone **7a** (0.2 mmol), morpholine **8a** (0.3 mmol), **PC1** (1.0 mol %), NiBr_2_·diglyme (5.0 mol %), dmbpy (5.0 mol %) and **PS1** (1.0 equiv) in DMF under N_2_, irradiation with a 427 nm LED lamp at 20 °C for 2 hours. ^b^Isolated yield of **9a** after flash column chromatography. ^c^No reaction.

Attempts to increase the temperature of the process resulted in a complex reaction mixture in which only traces of the desired enaminone **9a** were detected (Table 1, entry 12). Longer reaction times also led to significant degradation, isolating the desired enaminone in only 18% yield (Table 1, entry 13). The reaction with 3-chlorochromone gives lower yield while 3-iodochromone failed to provide the desired enaminone **9a** (Table S7, Supporting Information File 1).

Control experiments including the reaction in the absence of visible-light or photocatalyst, showed no product formation (Table 1, entries 16 and 17). Interestingly, the yield of **9a** dropped to 30% in the absence of Ni salt and 33% in the absence of the pyridinium salt (Table 1, entries 14 and 15).

**Scheme 2:**
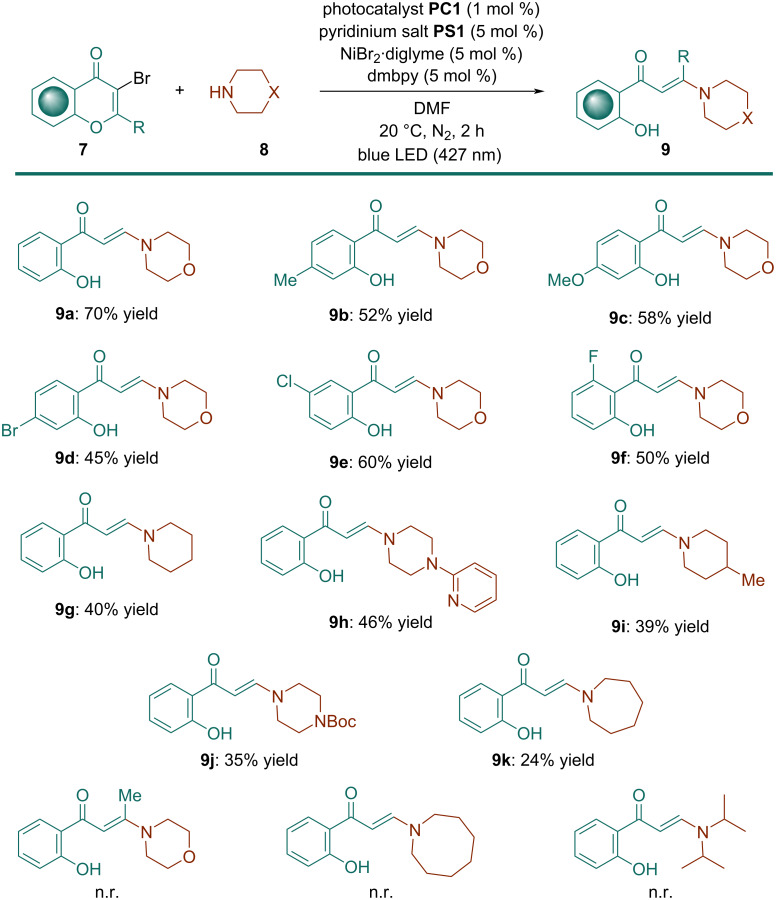
Substrate scope.

With the optimal reaction conditions, we first studied the substrate scope of 3-bromochromones **7** (Scheme 2).

It was determined that the process was compatible with various halochromones bearing electron-withdrawing (F, Cl, Br) or electron-donating (Me, OMe) groups at different positions of the aromatic ring, affording the corresponding enaminone products **9a**−**h** in moderate to good yields (45−70%). Of particular relevance is the effective formation of enaminones **9d** and **9e** containing bromo and chloro moieties, respectively, since they could enable further chemical transformations. Unfortunately, when 2-methyl-3-bromochromone was employed as the substrate, the process failed to afford the desired enaminone product, which was possibly caused by a combination of increased steric hindrance and decreased electrophilicity of the β-carbon due to the electron-donating nature of the methyl group.

Subsequently, we turned our attention to investigate a range of amine derivatives **8** under the standard conditions. When morpholine was replaced by piperidine, the expected enaminone **9g** was provided, albeit in lower yield. Similarly, 4*-*methylpiperidine and *N-tert*-butoxycarbonylpiperazine afforded the corresponding enaminones **9i** and **9j**, respectively, in moderate yields. Gratifyingly, 1-(pyridin-2-yl)piperazine was also tolerated, providing the expected product **9h** in 46% yield. Considering that pyridine is one of the core components of drug derivative formulations, present in more than 7,000 active pharmaceutical compounds [43], the possibility of introducing a pyridine ring into the enaminone structure could be useful for the development of new drug candidates.

When pyrrolidine was used as amine reagent, the target enaminone **9k** was obtained, albeit in low yield. Regrettably, this transformation failed to provide the corresponding enaminone products by replacing alicyclic amines by diisopropylamine, probably due to steric hindrance. Steric factors could also explain the lack of reactivity of seven-membered azepane. In view of these results, it seems evident that six-membered cyclic amines have the optimal ring size for the photocatalyzed enaminone formation process. For enaminones **9b**–**k** the remaining mass balance comprised mainly unreacted starting materials.

**Scheme 3:**
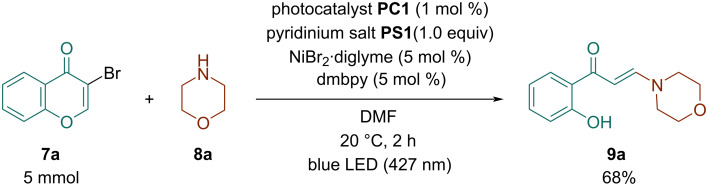
Scale-up synthesis of enaminone **9a**.

The scalability of the process was demonstrated in semipreparative scale for the reaction of 3-bromochromone (**7a**, 5.0 mmol) to afford enaminone **9a** in a 68% isolated yield (Scheme 3).

**Scheme 4:**
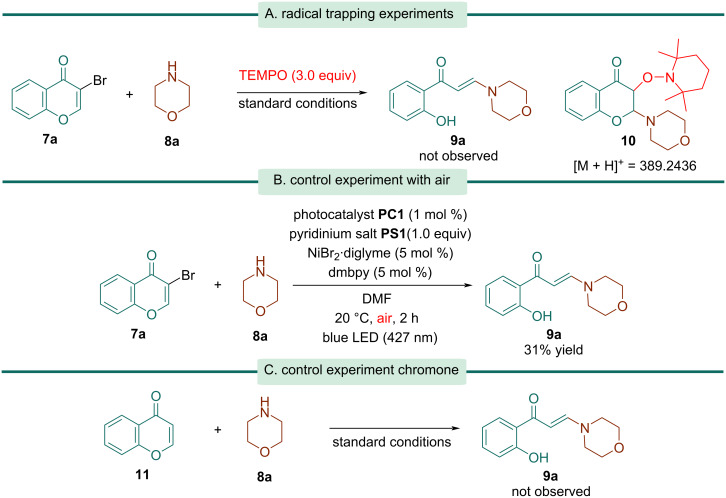
Mechanistic studies.

In terms of the reaction mechanism, TEMPO completely inhibited the reaction, implying the possibility of a radical intermediate in the reaction (Scheme 4A). Moreover, the TEMPO adduct **10** was identified using GC–MS (Figure S1, Supporting Information File 1). When the reaction was performed under air-equilibrated conditions, the intended product **9a** was obtained in a 31% yield, indicating that air influenced the interaction between the Ni-catalyst and the α,β-unsaturated carbonyl function (Scheme 4B). Furthermore, when the reaction of chromone **10** was carried out under standard conditions, the starting material was recovered unaltered, evidencing that the photocatalyzed dehalogenation step is crucial to enable the ring opening (Scheme 4C).

In order to determine the role of Ni(II) in this process, UV–vis studies were carried out. Thus, upon addition of **PS1** and **7a** to a solution of NiBr_2_-dmbpy, a charge transfer (CT) band at 688 nm was visible in the UV–vis spectrum (Figure S2, Supporting Information File 1). This observation is consistent with the formation of a six-coordinate, pseudo-octahedral Ni(II) complex [44] involving the carbonyl groups of **PS1** and **7a**, which is further activated on aggregation of the pyridinium salt and the chromone aromatic ring through π−π stacking.

Then, **PC1** was submitted to Stern–Volmer quenching experiments. Whereas no interaction occurred between the excited form of **PC1** and 3-bromochromone (**7a**), a direct interaction occurred between PC1* and morpholine (**8a**, Figure S3, Supporting Information File 1). Based on these results, the most plausible scenario might be that the reaction starts with the excitation of **PC1** at 427 nm to generate the highly oxidative state PC1* that interacts with morpholine (**8a**) to generate the corresponding aminium radical cation.

To gain a better understanding of the process, the formation of the enaminone product **9a** was monitored overtime by ^1^H NMR, which confirmed that the the reaction was complete within 2 h. Furthermore, the rate of the reaction is independent of the concentration of the 3-bromochromone substrate **7a** and amine **8a** (Figure S4, Supporting Information File 1).

**Scheme 5:**
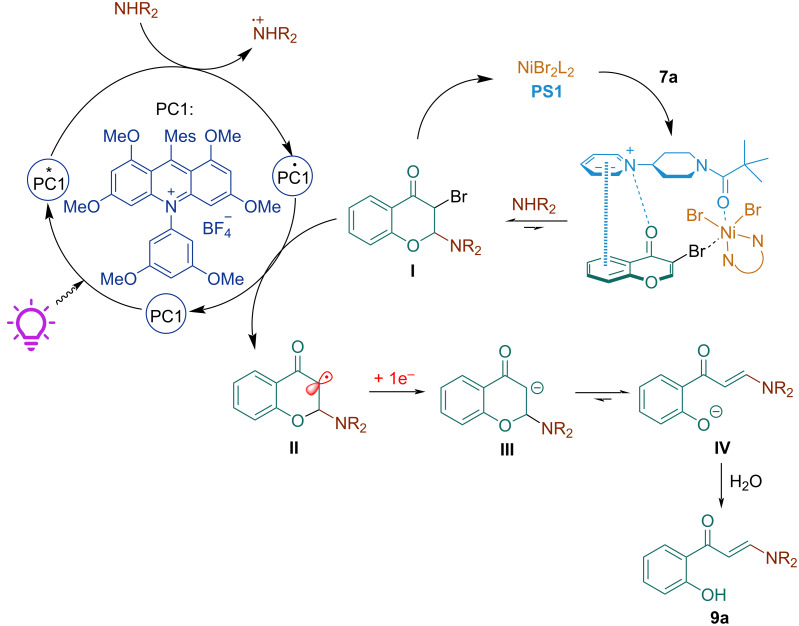
Proposed mechanism.

Based on the above experiments and prior work on the photocatalytic reductive halogenation using acridinium photocatalysts [45–46], a possible mechanism for this reaction is proposed in Scheme 5.

A ternary complex is initially formed upon complexation of 3-bromochromone (**7a**) with NiBr_2_-dmbpy. By virtue of being coordinated to a Ni-center, the β-carbon is activated toward nucleophilic attack of the amine, furnishing 2-amino-3-bromochromenone **I**. Simultaneously, acridinium photocatalyst **PC1** absorbed energy and transitioned from the ground state to excited state under visible-light irradiation. This excited state PC1* is quenched by the amine, generating the amine radical cation and **PC1** radical via a single-electron transfer (SET) process. Then, the C−Br bond of **I** is cleaved by **PC1**^•^, generating a C-centered radical **II**, which can be further reduced to give an enolate **III**, that ultimately evolves to the more stable anion **IV** and undergoes protonation to afford the final enaminone product **9a**.

## Conclusion

In summary, a simple and effective nickel-assisted photocatalytic protocol for the direct formation of enaminones from 3-bromochromones is reported. The process involves a Ni(II)-catalyzed hydroamination, followed by photocatalytic reductive debromination. Mechanistic studies suggest that the key step of this transformation is the complexation of the starting 3-bromochromones to Ni(II) and a pyridinium salt, giving rise to a ternary Ni-complex which activates the α,β-unsaturated carbonyl compound towards nucleophilic addition. The presented method is operationally simple and can be conducted using low catalyst loadings of a Ni-catalyst and an inexpensive organic photocatalyst.

## Supporting Information

File 1 Additional optimization details, mechanistic studies, experimental details, characterization data and NMR spectra for enaminones **9**.

## Data Availability

All data that supports the findings of this study is available in the published article and/or the supporting information of this article.
