# Supplementary material for: General method for the synthesis of enaminones via photocatalysis
Source: Beilstein J Org Chem. 2025 Jul 29;21:1535–43. doi: 10.3762/bjoc.21.116 (PMC12320736; doi:10.3762/bjoc.21.116)
Supplement: File 1 — Additional optimization details, mechanistic studies, experimental details, characterization data and NMR spectra for enaminones 9. [file Beilstein_J_Org_Chem-21-1535-s001.pdf]

BEILSTEIN JOURNAL OF ORGANIC CHEMISTRY

Supporting Information

for

General method for the synthesis of enaminones via photocatalysis

Paula Pérez-Ramos, Raquel G. Soengas and Humberto Rodríguez-Solla

Beilstein J. Org. Chem. **2025,** *21,* 1535–1543. doi:10.3762/bjoc.21.116

Additional optimization details, mechanistic studies, experimental details, characterization data and NMR spectra for enaminones 9

License and Terms: This is a supporting information file under the terms of the Creative Commons Attribution License (https://creativecommons.org/licenses/by/4.0). Please note that the reuse, redistribution and reproduction in particular requires that the author(s) and source are credited and that individual graphics may be subject to special legal provisions.
The license is subject to the *Beilstein Journal of Organic Chemistry* terms and conditions: (https://www.beilstein-journals.org/bjoc/terms)

CONTENTS

1. Additional optimization details…………………………………………..…………..…S2

2. Mechanistic studies……………………….....……………………..………………..….S6

3. Experimental details and characterization data..…………………….…………….S10

4. NMR spectra for enaminones **9**…………...……………………..…………..……...S14

5. References…………....…………………………………..…..………….....…….S25

1. Additional optimization details

Because of limited space, some details of optimization of the enaminone formation were omitted from the main text. The full optimization data are reported here.

Table S1: Solvent effect.

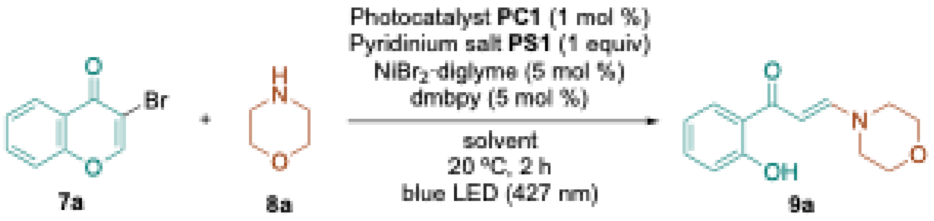

Entry	Solvent	Yield (%)
1	DMF	70
2	acetone	51
3	DMSO	55
4	DME	57
5	toluene	19
6	CH_2Cl_2	32

Table S2: Nickel salt and ligand**.**

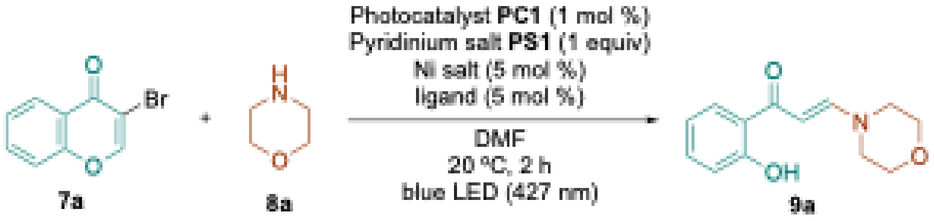

Entry	Ni salt	Ligand	Yield (%)
1	$NiBr_2$·diglyme	dmbpy	70
2	$Ni(OTf)_2$	dmbpy	62
3	$NiCl_2$·diglyme	dmbpy	49
4	$NiBr_2$·diglyme	dtbbpy	66
5	$NiBr_2$·diglyme	phenantroline	49

Table S3: Photocatalyst screening.

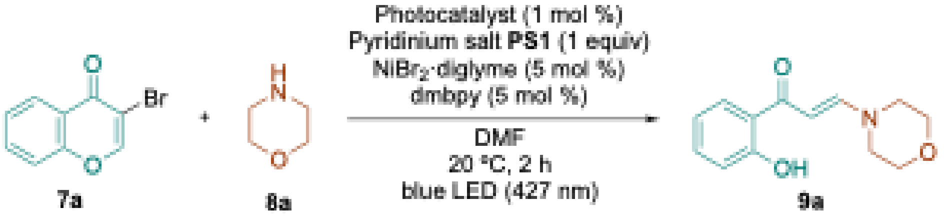

Entry	Photocatalyst (**PC**)	Yield (%)
1	10-(3,5-dimethoxyphenyl)-9-mesityl-1,3,6,8-tetramethoxyacridin-10-ium tetrafluoroborate (**PC1**)	70
2	4-CzIPN (**PC2**)	62[a]
3	$[Ir(dF(CF_3)ppy)_2(dtbbpy)]PF_6$ (**PC3**)	49
4	Eosin Y	n.r.[b]
5	$Ru(bpz)_3$	n.r.

[a] Reaction performed at 440 nm. [b] Reaction performed at 525 nm

Table S4: Walelenght and light intensity.

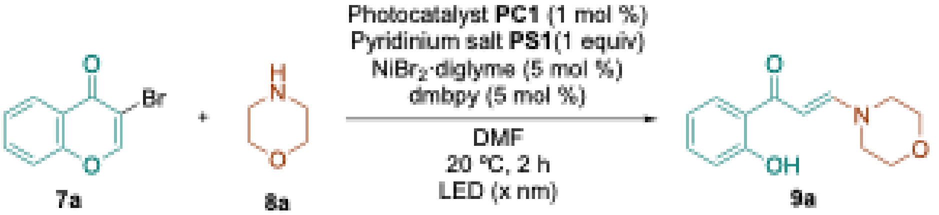

Entry	Wavelenghth	Yield (%)
1	427	70
2	427	69[a]
3	440	65
4	465	49
5	530	n.r.[b]

[a] Reaction performed with two lamps. [b] Reaction performed at 525 nm

Table S5: Pyridinium salt and stoichiometry.

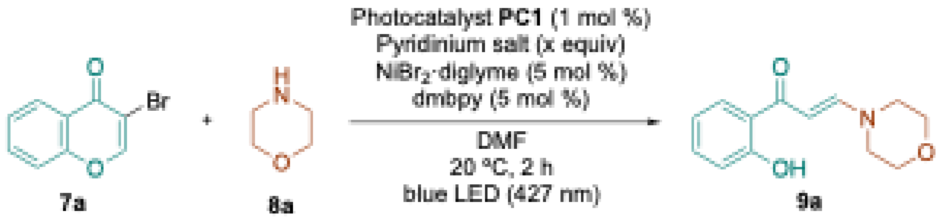

Entry	Pyridinium salt (**PS**)	equiv **PS**	equiv **8a**	Yield (%)
1	1-[1-(*tert*-butoxycarbonyl)piperidin-4-yl]-2,4,6-triphenylpyridin-1-ium (**PS1**)	1	1.5	70
2	1-benzyl-2,4,6-triphenylpyridin-1-ium (**PS2**)	1	1.5	34
3	1-[1-(*tert*-butoxycarbonyl)piperidin-4-yl]-2,4,6-triphenylpyridin-1-ium (**PS1**)	0.5	1.5	58
4	1-[1-(*tert*-butoxycarbonyl)piperidin-4-yl]-2,4,6-triphenylpyridin-1-ium (**PS1**)	1	1.0	61
5	1-[1-(*tert*-butoxycarbonyl)piperidin-4-yl]-2,4,6-triphenylpyridin-1-ium (**PS1**)	1	3.0	70

Table S6: Reaction time and temperature.

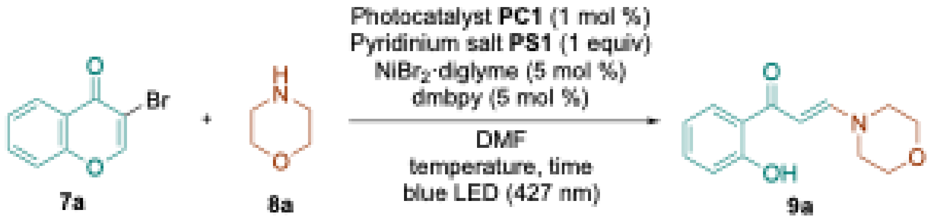

Entry	Time (h)	Temperature (ºC)	Yield (%)
1	2	20	70
2	2	40	18
3	2	0	42
4	6	1	61
5	16	1	18

Table S7: Halogen effect.

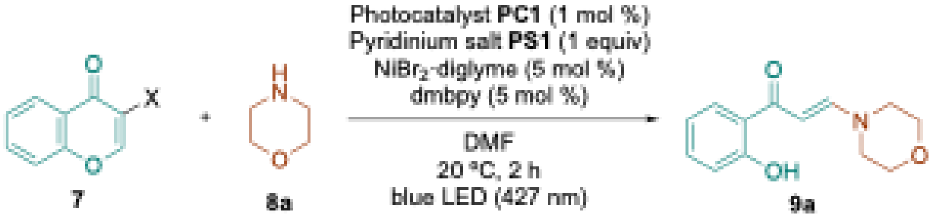

Entry	X	Yield (%)
1	Br	70
2	Cl	31
3	I	n.r.

Table S8: Individual optimization.

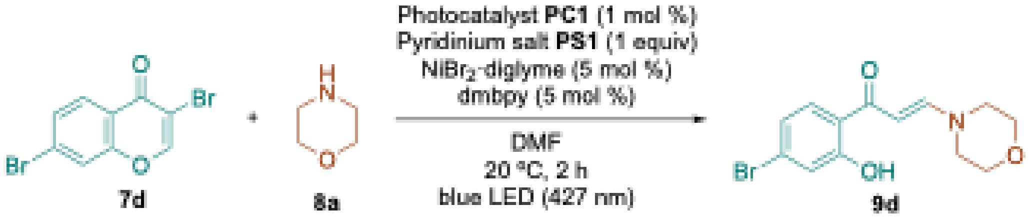

Entry	Time (h)	solvent	equiv **8a**	Yield (%)
1	2	DMF	1.5	45
2	2	DME	1.5	29
3	2	DMF	3.0	46
4	6	DMF	1.5	38

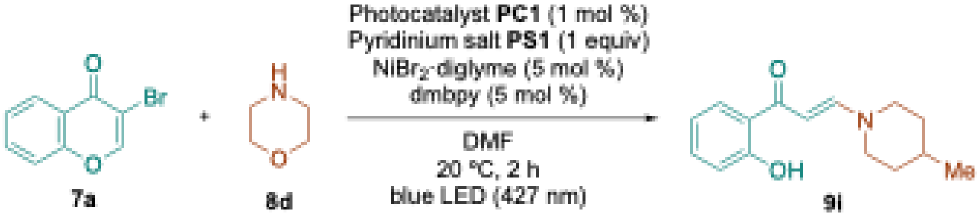

Entry	Time (h)	solvent	Yield (%)
1	2	DMF	39
2	2	DMF	39[a]
3	6	DMF	31

[a] Reaction performed with two lamps

2. Mechanistic studies

The identification of the TEMPO adduct was carried out using a high-resolution Quadrupole Time-of-flight mass spectrometry equipment coupled to a Gas Chromatograph (Agilent 7250) (GC-Q-TOF). UV-visible absorption spectra were obtained on a Cary 60 UV–vis spectrophotometer from Agilent Technologies (Palo Alto, CA, USA) by using a rectangular quartz cell (light path 10 × 10 mm, chamber volume: 3.5 mL). Excitation and emission luminescence spectra were obtained with a Varian Cary Eclipse (Agilent Technologies) with a semi-micro quartz fluorescence cell (light path: 10 × 4 mm, chamber volume 1400 µL, excitation filter: auto and emission filter: open, excitation and emission slit: 5 nm). The progress of the reaction was monitored by ^{1}H-NMR in $CDCl_3$ at 300 MHz using methyl 3,5-dinitrobenzoate as internal standard.

2.1. GC-Q-TOF spectrometry

Sample Information

**Sample Name**	PPR_555	**Data File Path**	D:\Projects\SILVIA\Masa Exacta\Data\Masa Exacta\MRM-003-072025\PPR_555.d
**Sample ID**		**Acq Time (Local)**	7/2/2025 1:29:21 PM (UTC+02:00)
**Instrument**	LC-IMSQTOF	**Acq Method Path**	D:\Projects\Silvia\Masa Exacta\Methods\FIA\Masa Exacta_FIA_sin ISO.m
**MS Type**	QTOF	**Acq SW Version**	6200 series TOF/6500 series Q-TOF (11.0.221.1)
**Inj Vol (ul)**	1	**IRM Status**	Success
**Sample Position**	P1-A1	**DA Method Path**	D:\MassHunter\Methods\12.0\Masa Exacta FIA.m
**Plate Position**		**Target Source Path**	
**Acq Operator**	SYSTEM (SYSTEM)	**Result Summary**	1 qualified (4 targets)

Sample Chromatograms

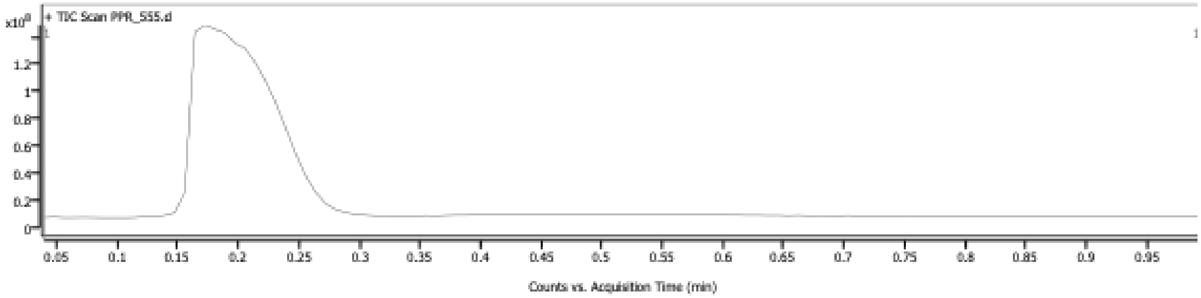

Compound Summary

Cpd	Name	Formula	CAS	RT	Mass	Mass (Tgt)	Diff (Tgt, ppm)	Score	Algorithm
1		C22 H32 N2 O4		0.164	388.2365	388.2362	0.82	80.32	FBF

Compound Details

Cpd 1: C22 H32 N2 O4

Name	Formula	RT	RI	Mass	Diff (Tgt, ppm)	CAS	ID Source	Score	Algorithm
	C22 H32 N2 O4	0.164		388.2365	0.82		FBF	80.32	FBF

Species	m/z	Score (Tgt)	Score (Lib)	Score (DB)	Score (MFG)	Score (RT)
(M+H)+	389.2436	80.32				

Compound Chromatograms (overlaid)

Structure

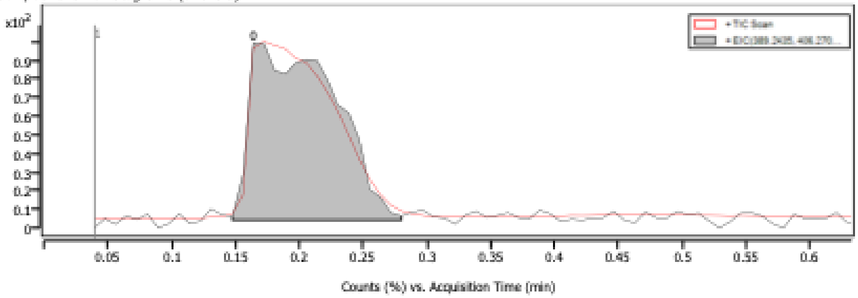

Compound Spectra (overlaid)

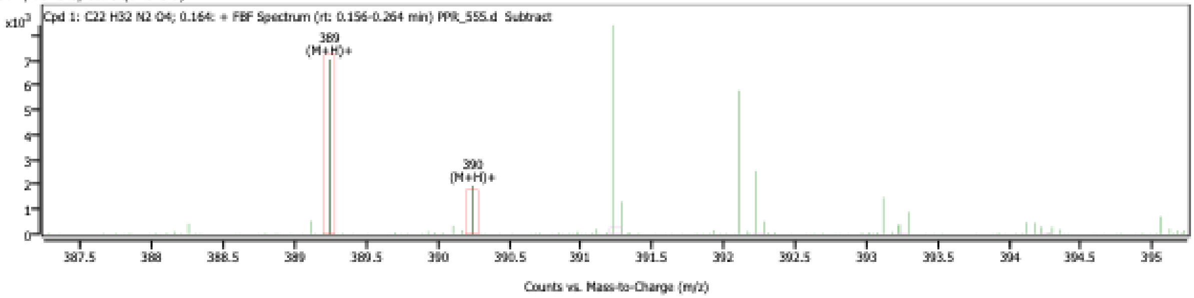

Figure S1: Identification of the TEMPO adduct of intermediate radical **I**.

2.2. UV–vis spectroscopy

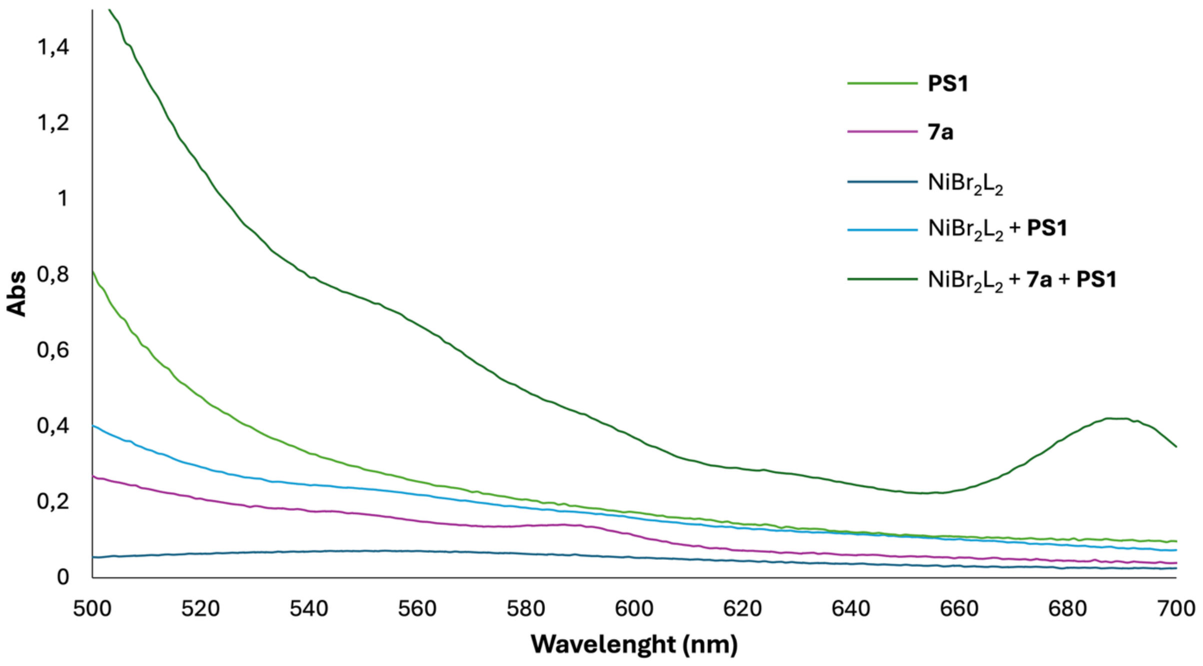

Figure S2: UV–vis studies.

2.3. Luminescence quenching experiments

Fluorescence quenching studies were carried out using a 5.0×10^{-6} M solution of **PC1** irradiated at 427 nm, with variable concentrations of morpholine in degassed DMF.

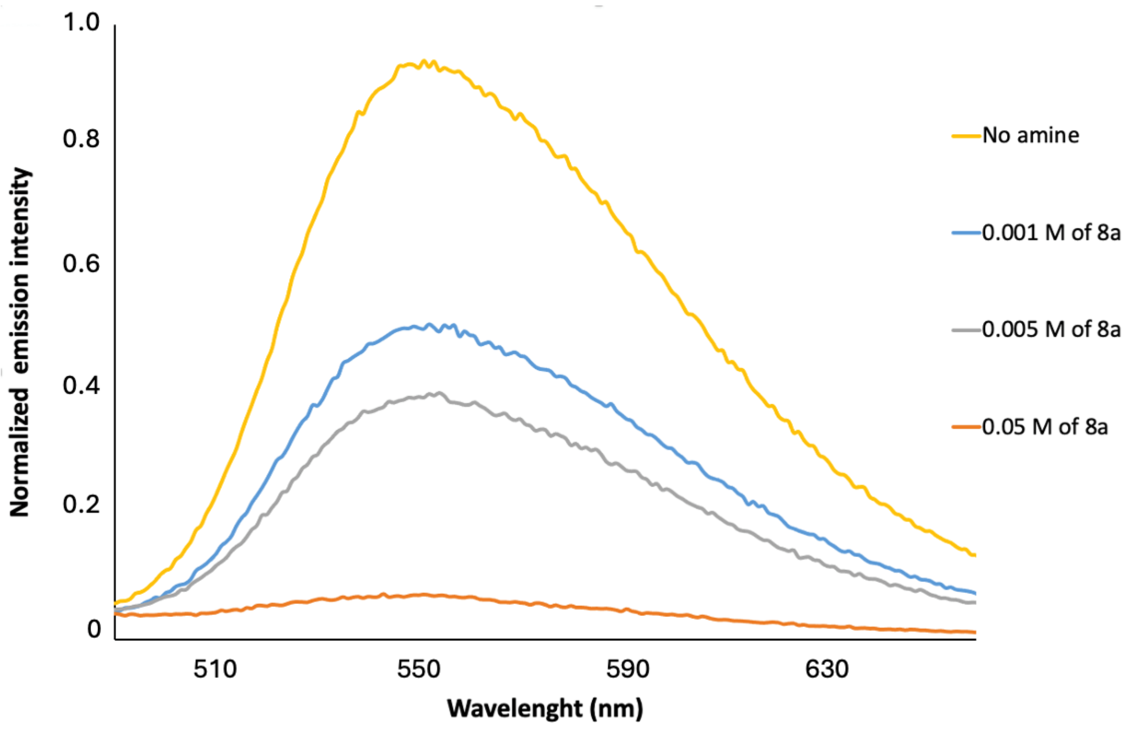

Figure S3: Fluorescence quenching of **PC1*** and morpholine.

2.4. Reaction progress analysis

The reaction of 3-bromochromone (**7a**) and morpholine (**8a**) to afford enaminone **9a** was carried out under the standard conditions and at varying concentrations of **7a**. At intervals of 15, 30, 60, 90 and 120 min, an aliquot was extracted from the reaction and, after aqueous work-up, analyzed by ^{1}H NMR (alkene signal at 5.91 ppm, methyl 3,5-dinitrobenzoate as internal standard).

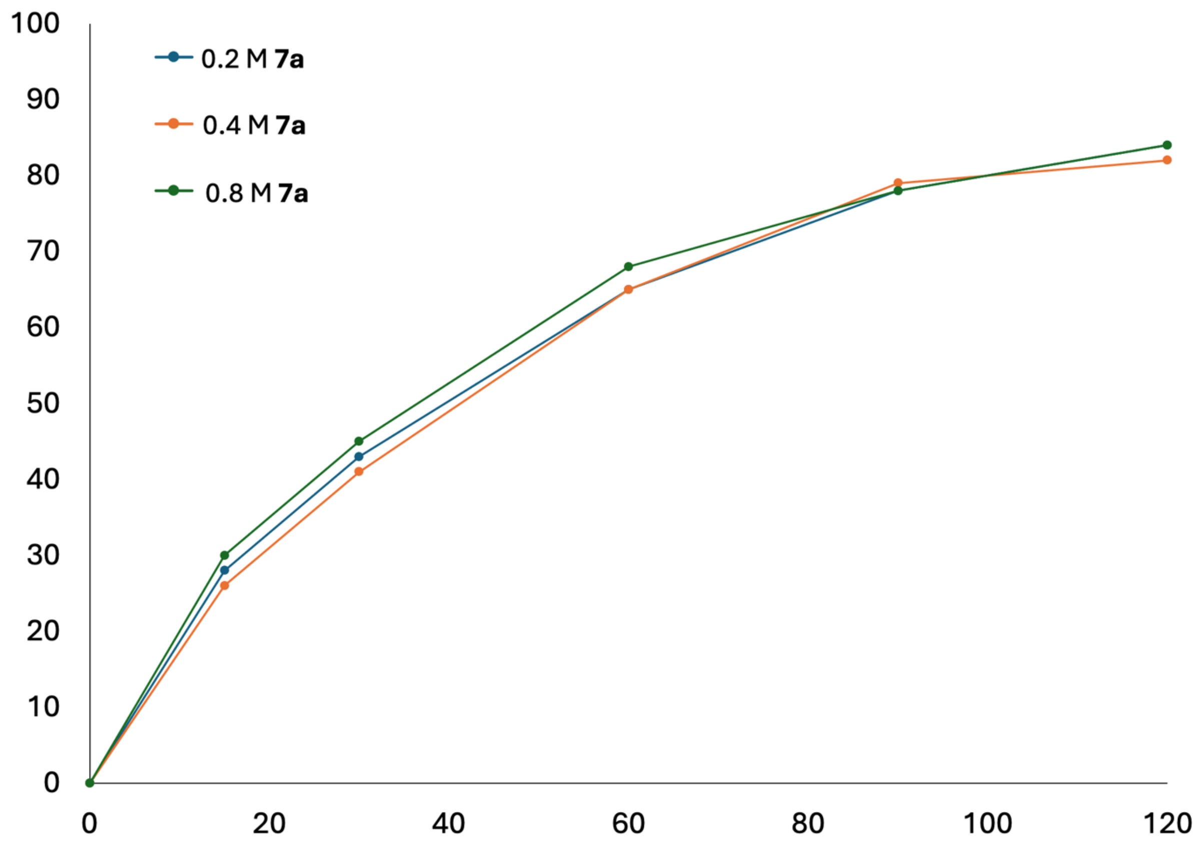

Figure S4: Monitoring of the formation of the enamine **9a** by ^{1}H NMR.

3. Experimental details and characterization data

3.1. General experimental procedures

All 3-bromochromones **7** were prepared following previously reported methodologies [1]. The different reagents employed during the development of this work are commercially available and were purchased from Chemosapiens S. L. Dry DMF stored over molecular sieves commercially available from Sigma Aldrich Chemical co. was used for the photochemical reactions. NMR spectra were recorded in $CDCl_3$ at 300 MHz for ^1H and 75 MHz for ^{13}C, with tetramethylsilane as internal standard for ^1H and the residual solvent signals as standard for ^{13}C. The data is being reported as s = singlet, bs = broad singlet, d = doublet, dd = double doublet, t = triplet, dt = double triplet, q = quatriplet, p = quintuplet and m = multiplet or unresolved, chemical shifts in ppm and coupling constant(s) in Hz. The values of the chemical shift of the signals in the NMR reports are in ppm. HRMS were measured in ESI mode, and the mass analyzer of the HRMS was TOF (Bruker model Impact II).

3.2. Photochemical reactions set-up

A Kessil® PR160 Rig equipped with a PR160-427nm lamp was used for the photochemistry setup. A glassware reactor developed in our group was used to run the photochemical reactions under controlled temperature. [2] The reactor was placed at approximately 5 cm away from the lamp prior to irradiation at maximum intensity (100% power) of the Kessil lamp.

3.3. Synthesis of enaminones 9

A solution of $NiBr_2$·diglyme (0.01 mmol) and dmbpy (0.01 mmol) in DMF (1 mL) was stirred for 5 min under N_2. Subsequently, was added 3-bromochromone **7** (0.2 mmol), amine **8** (0.3 mmol), **PC1** (0.002 mmol), **PS1** (0.2 mmol) and DMF (1.0 mL). Then the resulting mixture was stirred at 20 °C and irradiated with a 20 W 427 nm Kessil LEDs for 2 h. After the reaction was completed, the solvent was removed under reduced pressure, and the residue was extracted with ethyl acetate, washed with brine, and dried over anhydrous Na_2SO_4. The crude product was purified by flash column chromatography on silica gel using Hex/EtOAc (1:1) as the eluent to afford the title compounds **9**.

3.4. Characterization data of enaminones 9

(E)-1-(2-Hydroxyphenyl)-3-morpholinoprop-2-en-1-one ***(9a)*** [3]*:* 33 mg, 70% yield. Yellow solid. R_f = 0.42 (Hex/EtOAc 1:1); ^{1}H NMR (300 MHz, $CDCl_3$): δ 3.42 (t, *J* = 5.0 Hz, 4H, 2 x CH_2), 3.70 (t, *J* = 4.7 Hz, 4H, 2 x CH_2), 5.91 (d, *J* = 12.4 Hz, 1H, CH), 6.86-6.77 (m, 1H, ArH), 6.93 (dd, *J* = 8.3, 1.3 Hz, 1H, ArH), 7.35 (ddd, *J* = 8.6, 7.1, 1.7 Hz, 1H, ArH), 7.65 (dd, *J* = 8.1, 1.8 Hz, 1H, ArH), 7.80 (d, *J* = 12.4 Hz, 1H, CH), 13.78 (s, 1H, OH); ^{13}C NMR (75 MHz, $CDCl_3$): δ 46.7, 52.8, 66.2, 89.8, 118.17, 118.23, 120.3, 128.3, 134.3, 153.2, 162.5, 192.1. HRMS (ESI^+): *m/z* $[M]^+$ calcd. for $C_{13}H_{15}NO_3$, 233.2670; found: 233.2672.

(E)-1-(2-Hydroxy-4-methylphenyl)-3-morpholinoprop-2-en-1-one ***(9b)****:* 26 mg, 52% yield. Yellow solid. R_f = 0.45 (Hex/EtOAc 1:1); ^{1}H NMR (300 MHz, $CDCl_3$): δ 2.34 (s, 3H, CH_3), 3.45 (t, *J* = 4.9 Hz, 4H, 2 x CH_2), 3.79 (t, *J* = 4.9 Hz, 4H, 2 x CH_2), 5.92 (d, *J* = 12.4 Hz, 1H, CH), 6.65 (d, *J* = 8.1 Hz, 1H, ArH), 7.56 (d, *J* = 8.1 Hz, 1H, ArH), 7.65 (dd, *J* = 8.1, 1.8 Hz, 1H, ArH), 7.81 (d, *J* = 12.4 Hz, 1H, CH), 13.78 (s, 1H, OH); ^{13}C NMR (75 MHz, $CDCl_3$): δ 21.7, 66.2, 90.2, 118.5, 119.3, 127.1, 128.1, 145.5, 152.8, 163.0, 191.9. HRMS (ESI^+): *m/z* $[M]^+$ calcd. for $C_{14}H_{17}NO_3$, 247.1208; found: 247.1212.

(E)-1-(2-Hydroxy-4-methoxyphenyl)-3-morpholinoprop-2-en-1-one ***(9c)****:* 31 mg, 58% yield. Yellow solid. R_f = 0.35 (Hex/EtOAc 1:1); ^{1}H NMR (300 MHz, $CDCl_3$): δ 3.42 (t, *J* = 5.0 Hz, 4H, 2 x CH_2), 3.77 (t, *J* = 4.8 Hz, 4H, 2 x CH_2), 3.82 (s, 3H, CH_3), 5.84 (d, *J* = 12.4 Hz, 1H, CH), 6.44-6.36 (m, 2H, ArH), 7.59 (d, *J* = 8.9 Hz, 1H, ArH), 7.77 (d, *J* = 12.4 Hz, 1H, CH), 14.32 (s, 1H, OH); ^{13}C NMR (75 MHz, $CDCl_3$): δ 55.4, 66.3, 90.0, 101.0, 106.6, 113.81, 129.7, 152.5, 164.6, 165.6, 191.1. HRMS (ESI^+): *m/z* $[M]^+$ calcd. for $C_{14}H_{17}NO_4$, 263.1158; found: 263.1157.

(E)-1-(4-Bromo-2-hydroxyphenyl)-3-morpholinoprop-2-en-1-one ***(9d)****:* 28 mg, 45% yield. Yellow solid. R_f = 0.43 (Hex/EtOAc 7:3); ^{1}H NMR (300 MHz, $CDCl_3$): δ 3.46 (t, *J* = 5.0 Hz, 4H, 2 x CH_2), 3.79 (t, *J* = 4.9 Hz, 4H, 2 x CH_2), 5.84 (d, *J* = 12.3 Hz, 1H, CH), 6.92 (dd, *J* = 8.6, 2.0 Hz, 1H, ArH), 6.95 (d, *J* = 2.0 Hz, 1H, ArH), 7.49 (d, *J* = 8.6 Hz, 1H, ArH), 7.82 (d, *J* = 12.3 Hz, 1H, CH), 13.98 (s, 1H, OH); ^{13}C NMR (75 MHz, $CDCl_3$): δ 45.9, 53.5, 65.7, 89.7, 119.8, 121.0, 122.7, 127.6, 133.9, 153.7, 161.4, 190.8. HRMS (ESI^+): *m/z* $[M]^+$ calcd. for $C_{13}H_{14}BrNO_3$, 311.0157; found: 311.0172.

(E)-1-(5-Chloro-2-hydroxyphenyl)-3-morpholinoprop-2-en-1-one ***(9e)*** [4]*:* 32 mg, 60% yield. Yellow solid. R_f = 0.12 (Hex/EtOAc 1:1); ^{1}H NMR (300 MHz, $CDCl_3$): δ 3.49 (t, *J*

= 5.7 Hz, 4H, 2 x CH_2), 3.79 (t, *J* = 4.2 Hz, 4H, 2 x CH_2), 5.84 (d, *J* = 12.2 Hz, 1H, CH), 6.93-6.88 (m, 1H, ArH), 7.30 (dd, *J* = 8.8, 2.3 Hz, 1H, ArH), 7.61 (t, *J* = 2.6 Hz, 1H, ArH), 7.85 (d, *J* = 12.3 Hz, 1H, CH), 13.76 (s, 1H, OH); ^{13}C NMR (75 MHz, $CDCl_3$): δ 46.0, 53.5, 66.6, 89.7, 119.8, 121.0, 122.6, 127.6, 133.9, 153.7, 161.4, 190.8. HRMS (ESI$^+$): *m/z* [M]$^+$ calcd. for $C_{13}H_{14}ClNO_3$, 267.0662; found: 267.0667.

*(E)-1-(2-Fluoro-6-hydroxyphenyl)-3-morpholinoprop-2-en-1-one (**9f**):* 25 mg, 50% yield. Yellow solid. R_f = 0.40 (Hex/EtOAc 1:1); ^{1}H NMR (300 MHz, $CDCl_3$): δ 3.45 (bs, 4H, 2 x CH_2), 3.77 (t, *J* = 4.9 Hz, 4H, 2 x CH_2), 6.04 (d, *J* = 12.4 Hz, 1H, CH), 6.51 (ddd, *J* = 12.3, 8.3, 1.2 Hz, 1H, ArH), 6.72 (d, *J* = 8.3 Hz, 1H, ArH), 7.20–7.28 (m, 1H, ArH), 7.92 (dd, *J* = 12.3, 3.0 Hz, 1H, CH), 14.23 (s, 1H, OH); ^{13}C NMR (75 MHz, $CDCl_3$): δ 46.0, 55.1, 67.4, 96.1 (d, *J* = 18.9 Hz), 105.6 (d, *J* = 25.7 Hz), 110.4 (d, *J* = 13.0 Hz), 114.1 (d, *J* = 2.8 Hz), 133.5 (d, *J* = 13.0 Hz), 153.9, 162.4 (d, *J* = 252.3 Hz), 164.5 (d, *J* = 5.4 Hz), 189.4 (d, *J* = 4.5 Hz). HRMS (ESI$^+$): *m/z* [M]$^+$ calcd. for $C_{13}H_{14}FNO_3$, 251.0954; found: 251.0958.

*(E)-1-(2-Hydroxyphenyl)-3-(piperidin-1-yl)prop-2-en-1-one (**9g**)* [5]: 18 mg, 40% yield. Yellow solid. R_f = 0.58 (Hex/EtOAc 1:1); ^{1}H NMR (300 MHz, $CDCl_3$): δ 1.71 (s, 6H, 3 x CH_2), 3.44 (s, 4H, 2 x CH_2), 5.90 (d, *J* = 12.2 Hz, 1H, CH), 6.82 (t, *J* = 7.6 Hz, 1H, ArH), 6.95 (d, *J* = 8.3 Hz, 1H, ArH), 7.36 (t, *J* = 7.7 Hz, 1H, ArH), 7.69 (d, *J* = 8.0 Hz, 1H, ArH), 7.88 (d, *J* = 12.2 Hz, 1H, CH), 14.04 (s, 1H, OH); ^{13}C NMR (75 MHz, $CDCl_3$): δ 25.0, 26.5, 46.6, 55.5, 80.0, 117.9, 118.2, 120.5, 128.1, 133.9, 153.5, 162.9, 191.7. HRMS (ESI$^+$): *m/z* [M]$^+$ calcd. for $C_{17}H_{17}NO_2$, 231.1259; found: 231.1257.

*(E)-1-(2-Hydroxyphenyl)-3-[4-(pyridin-2-yl)piperazin-1-yl]prop-2-en-1-one (**9h**):* 28 mg, 46% yield. Yellow solid. R_f = 0.52 (Hex/EtOAc 1:1); ^{1}H NMR (300 MHz, $CDCl_3$): δ 3.55 (t, *J* = 5.5 Hz, 4H, 2 x CH_2), 3.68 (dd, *J* = 6.3, 4.3 Hz, 4H, 2 x CH_2), 5.96 (d, *J* = 12.4 Hz, 1H, CH), 6.67-6.74 (m, 2H, ArH), 6.83 (ddd, *J* = 8.4, 7.2, 1.3 Hz, 1H, ArH), 6.94 (dd, *J* = 8.3, 1.3 Hz, 1H, ArH), 7.36 (ddd, *J* = 8.6, 7.1, 1.7 Hz, 1H, ArH), 7.53 (ddd, *J* = 8.9, 7.1, 2.0 Hz, 1H, ArH), 7.71 (dd, *J* = 8.1, 1.8 Hz, 1H, ArH), 7.90 (d, *J* = 12.4 Hz, 1H, CH), 8.21 (dd, *J* = 5.0, 2.2 Hz, 1H, ArH), 13.84 (s, 1H, OH); ^{13}C NMR (75 MHz, $CDCl_3$): δ 45.2, 53.0, 90.1, 107.3, 114.3, 118.1, 118.3, 120.9, 128.3, 134.2, 137.9, 148.1 153.1, 158.6, 162.9, 192.0. HRMS (ESI$^+$): *m/z* [M]$^+$ calcd. for $C_{18}H_{19}N_3O_2$, 309.1477; found: 309.1484.

*(E)-1-(2-Hydroxyphenyl)-3-(4-methylpiperidin-1-yl)prop-2-en-1-one (**9i**):* 19 mg, 39% yield. Yellow solid. R_f = 0.72 (Hex/EtOAc 1:1); ^{1}H NMR (300 MHz, $CDCl_3$): δ 1.05-0.97 (m, 3H, CH_3), 1.65-1.82 (m, 5H, CH, 2 x CH_2), 3.00, 3.34, 3.59, 3.81 (4 x bs, 4H, 2 x CH_2), 5.91 (dt, *J* = 12.1, 3.3 Hz, 1H, CH), 6.82 (td, *J* = 8.0, 4.3 Hz, 1H, ArH), 6.94 (dd, *J* = 7.8, 3.7 Hz, 1H, ArH), 7.36 (t, *J* = 8.8 Hz, 1H, ArH), 7.74-7.65 (m, 1H, ArH), 7.89 (dt, *J* = 12.3, 3.3 Hz, 1H, CH), 14.03 (s, 1H, OH); ^{13}C NMR (75 MHz, $CDCl_3$): δ 28.4, 34.1, 46.0, 53.8, 66.2, 90.2, 118.17, 118.23, 120.3, 128.3, 134.3, 153.2, 162.5, 192.1. HRMS (ESI$^+$): *m/z* [M]$^+$ calcd. for $C_{15}H_{19}NO_2$, 245.1416; found: 245.1418.

*tert-Butyl (E)-4-[3-(2-hydroxyphenyl)-3-oxoprop-1-en-1-yl]piperazine-1-carboxylate (**9j**):* 23 mg, 35% yield. Yellow solid. R_f = 0.69 (Hex/EtOAc 1:1); ^{1}H NMR (300 MHz, $CDCl_3$): δ 1.48 (s, 9H, 3 x CH_3), 3.41 (t, *J* = 5.8 Hz, 4H, 2 x CH_2), 3.54 (dd, *J* = 6.7, 3.8 Hz, 4H, 2 x CH_2), 5.93 (d, *J* = 12.5 Hz, 1H, CH), 6.82 (ddd, *J* = 8.0, 6.9, 1.2 Hz, 1H, ArH), 6.93 (dd, *J* = 8.3, 1.1 Hz, 1H, ArH), 7.36 (ddd, *J* = 8.6, 7.2, 1.7 Hz, 1H, ArH), 7.66 (dd, *J* = 8.1, 1.6 Hz, 1H, ArH), 7.84 (d, *J* = 12.6 Hz, 1H, CH), 13.74 (s, 1H, OH); ^{13}C NMR (75 MHz, $CDCl_3$): δ 28.3, 43.6, 49.7, 80.8, 90.4, 118.1, 118.3, 120.2, 128.3, 134.3, 153.1, 154.3, 162.9, 192.2. HRMS (ESI$^+$): *m/z* [M]$^+$ calcd. for $C_{18}H_{24}NO_2$, 332.4000; found: 332.4018.

*(E)-1-(2-Hydroxyphenyl)-3-(pyrrolidin-1-yl)-prop-2-en-1-one (**9k**):* 14 mg, 26% yield. Yellow solid. R_f = 0.56 (Hex/EtOAc 1:1); ^{1}H NMR (300 MHz, $CDCl_3$): δ 2.09 (p, *J* = 6.4 Hz, 2H), 2.17 (p, *J* = 6.4 Hz, 2H), 3.44 (t, *J* = 6.7 Hz, 2H), 3.71 (t, *J* = 6.7 Hz, 2H), 5.85 (d, *J* = 12.2 Hz, 1H, CH), 6.93 (t, *J* = 7.6 Hz, 1H, ArH), 7.05 (d, *J* = 7.6 Hz, 1H, ArH), 7.46 (t, *J* = 7.7 Hz, 1H, ArH), 7.81 (dd, *J* = 7.9, 1.4 Hz, 1H, ArH), 8.21 (d, *J* = 12.2 Hz, 1H, CH), 14.17 (s, 1H, OH); ^{13}C NMR (75 MHz, $CDCl_3$): δ 25.2, 47.2, 52.7, 90.9, 117.9, 118.2, 120.4, 128.2, 133.8, 150.5, 163.0, 191.2. HRMS (ESI$^+$): *m/z* [M]$^+$ calcd. for $C_{13}H_{15}NO_2$, 217.1103; found: 217.1109.

4. NMR spectra for enaminones 9

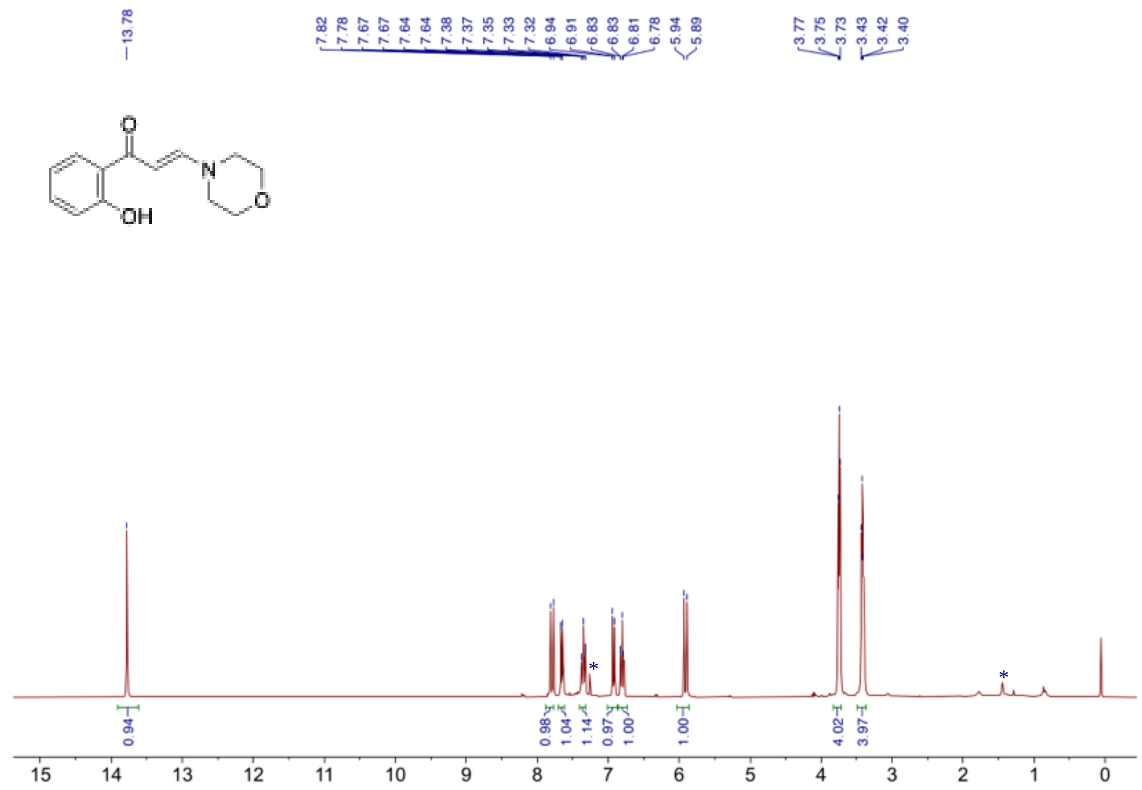

Figure S4: ^{1}H NMR spectra of **9a**.

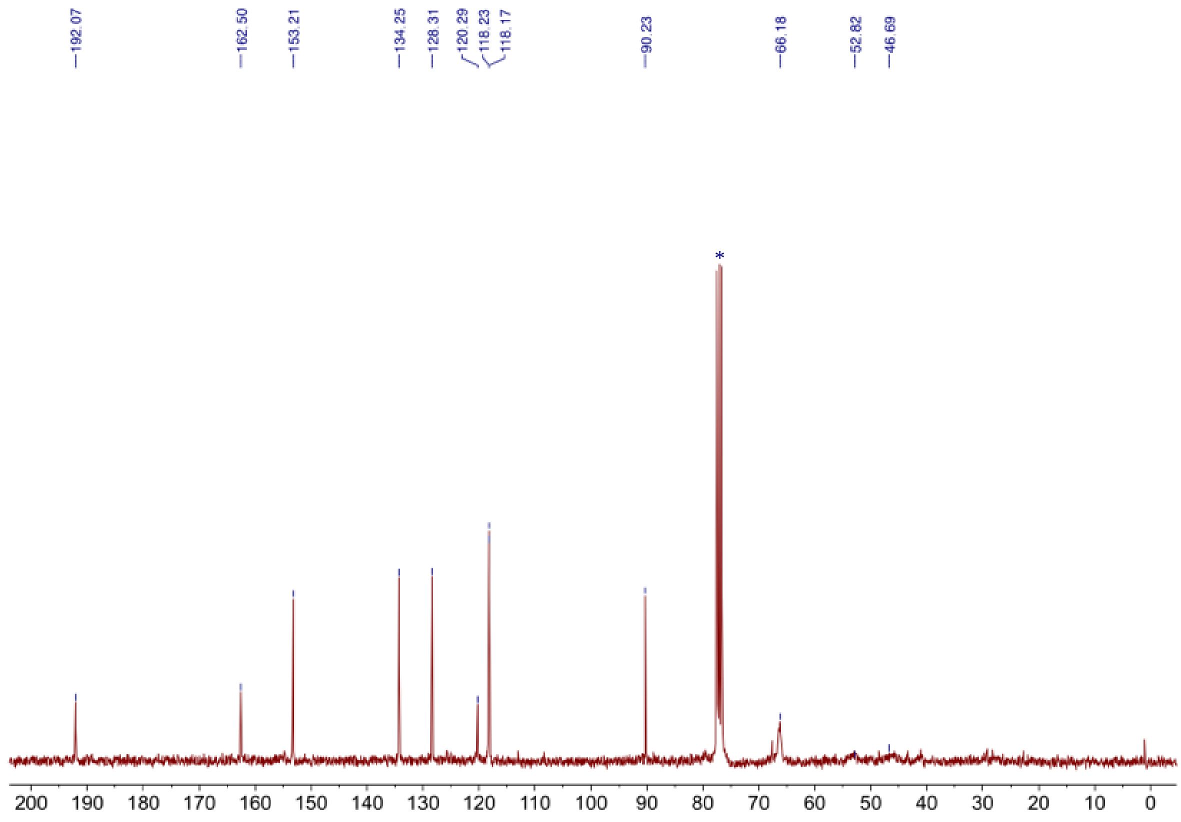

Figure S5: ^{13}C NMR spectra of **9a**.

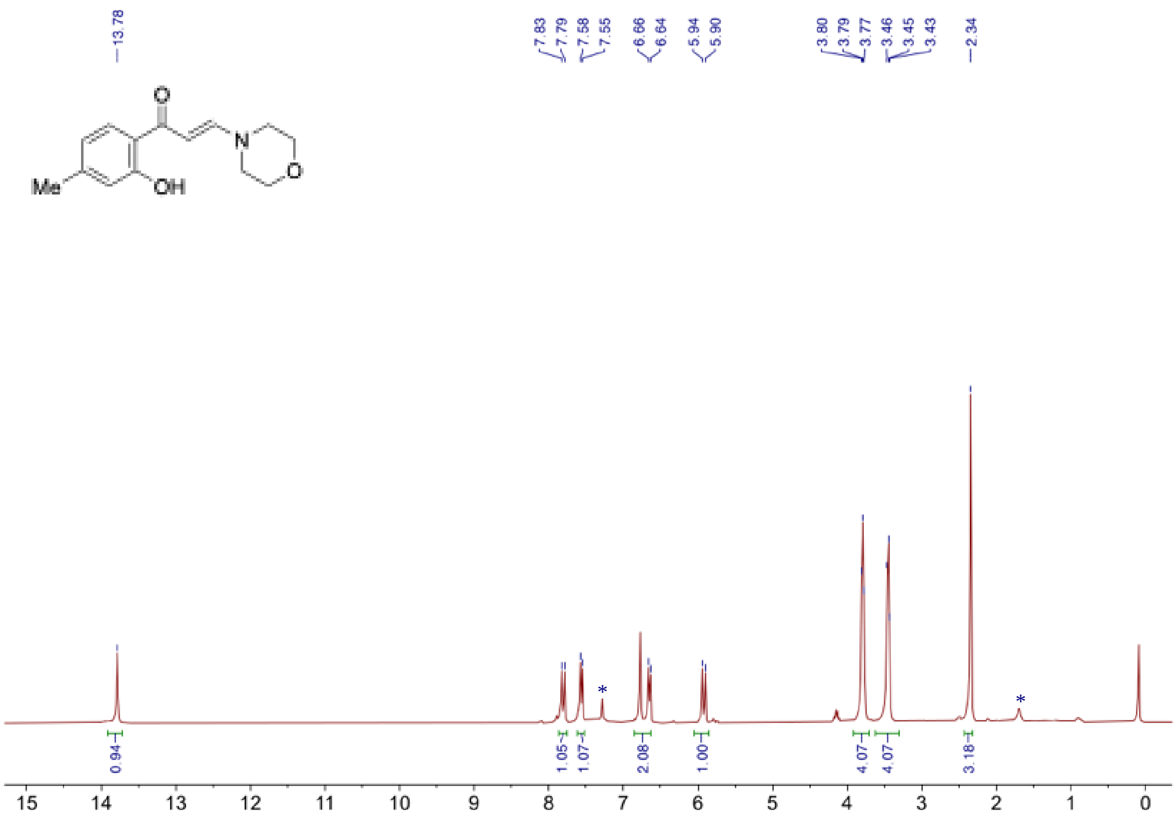

Figure S5: ^{1}H NMR spectra of **9b**.

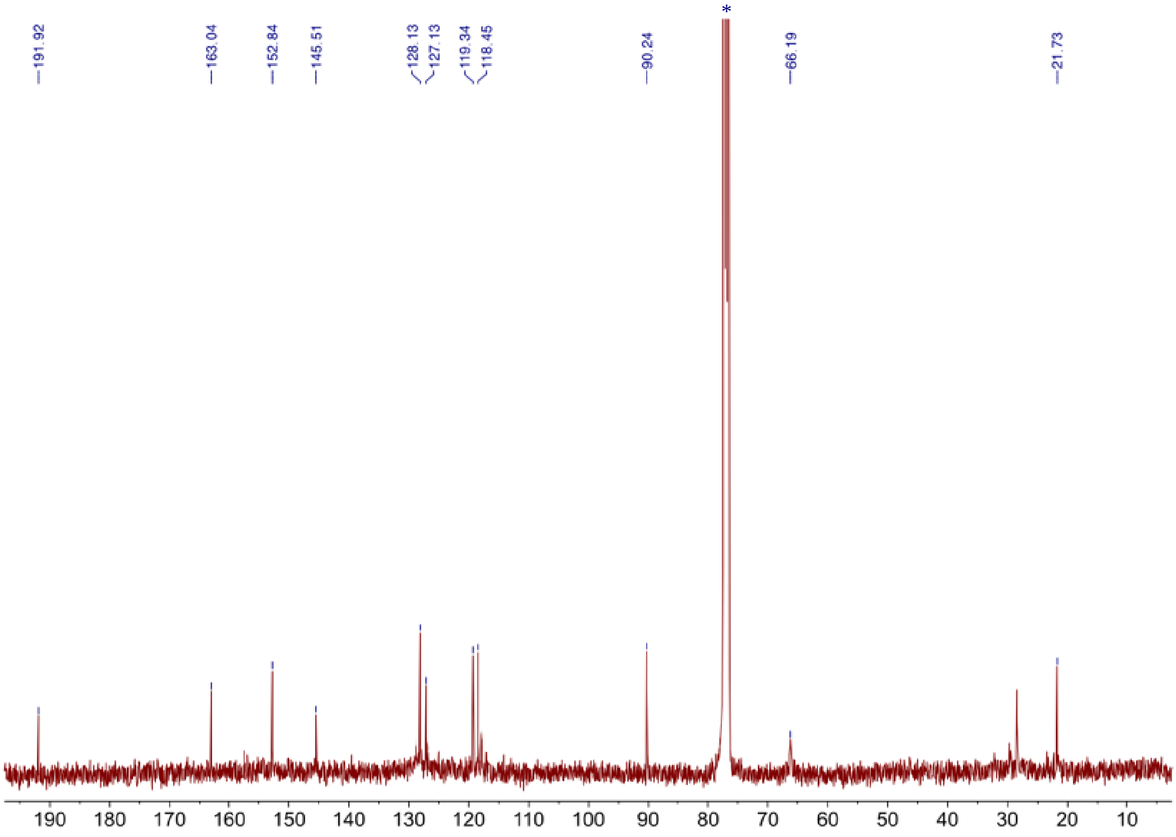

Figure S6: ^{13}C NMR spectra of **9b**.

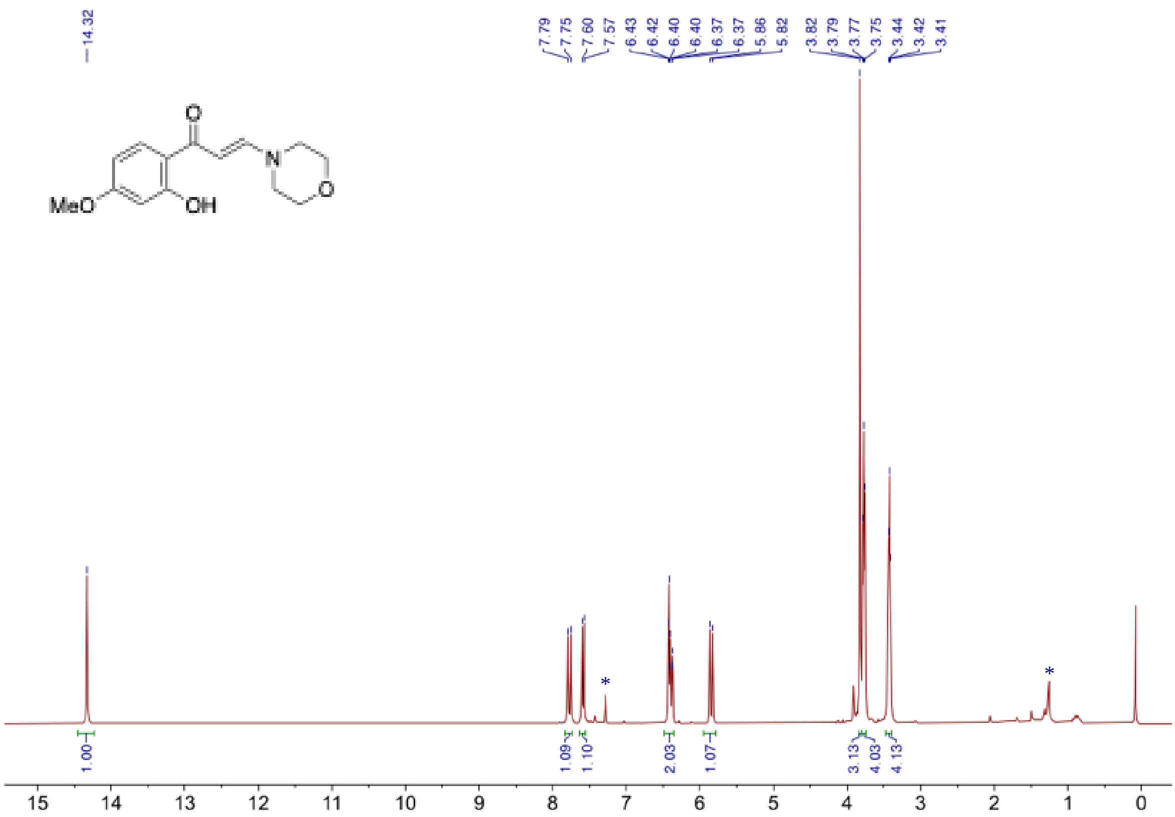

Figure S7: ^{1}H NMR spectra of **9c**.

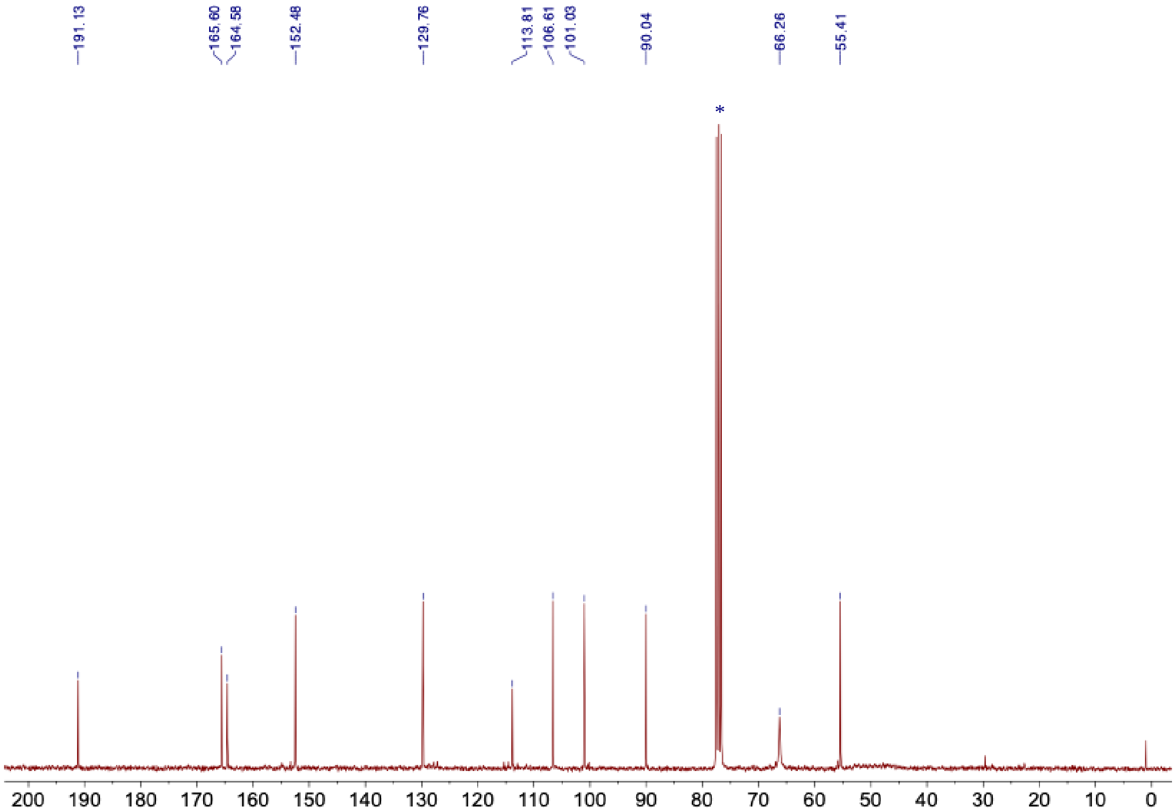

Figure S8: ^{13}C NMR spectra of **9c**.

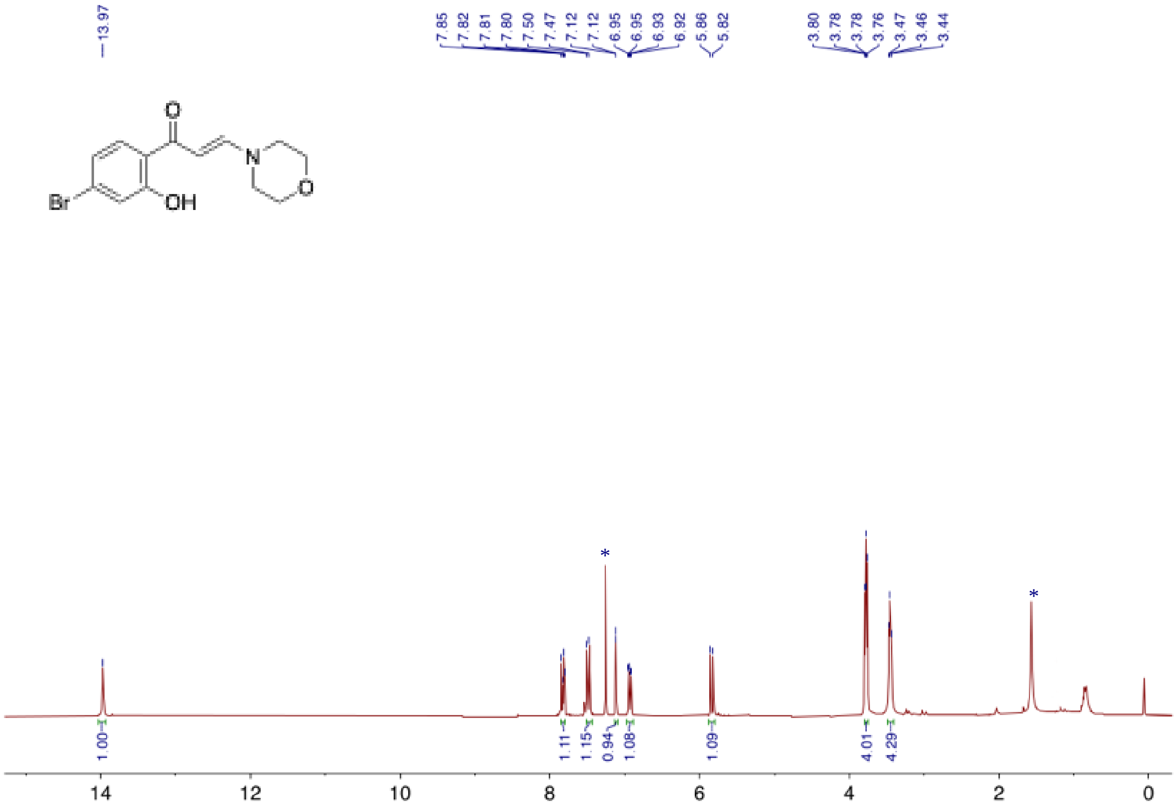

Figure S9: ^{1}H NMR spectra of **9d**.

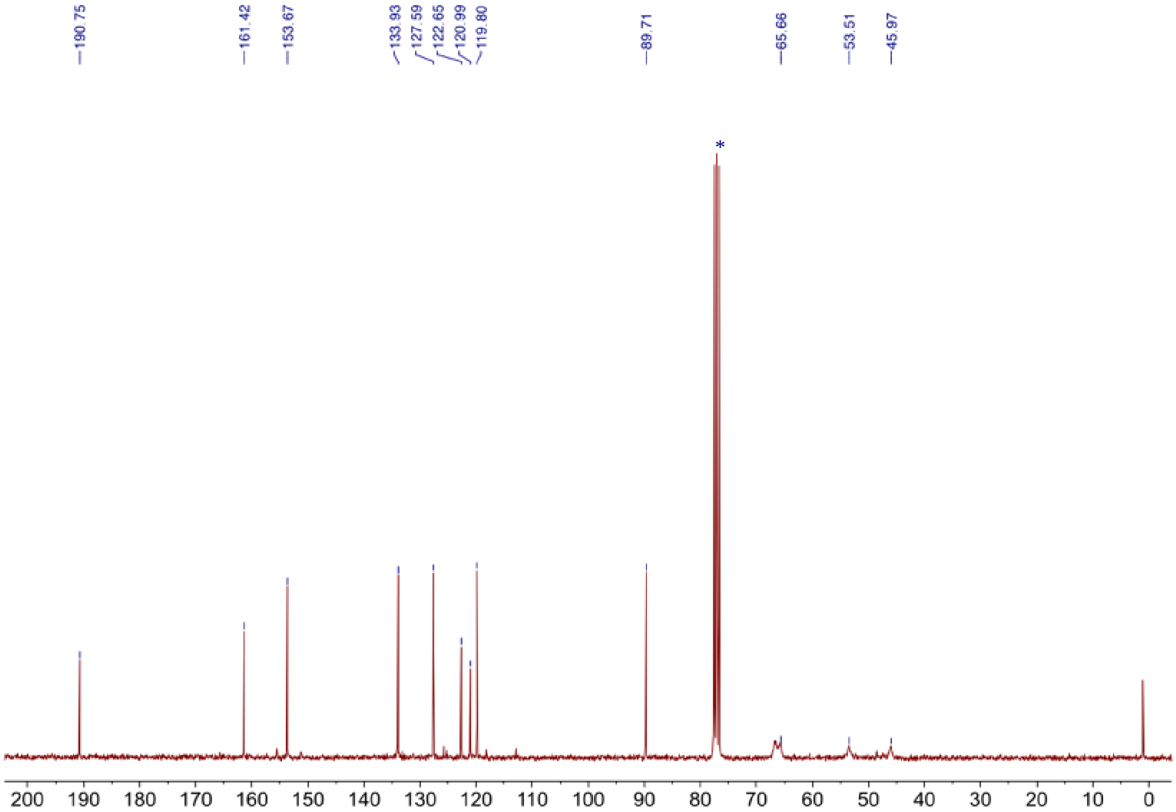

Figure S10: ^{13}C NMR spectra of **9d**.

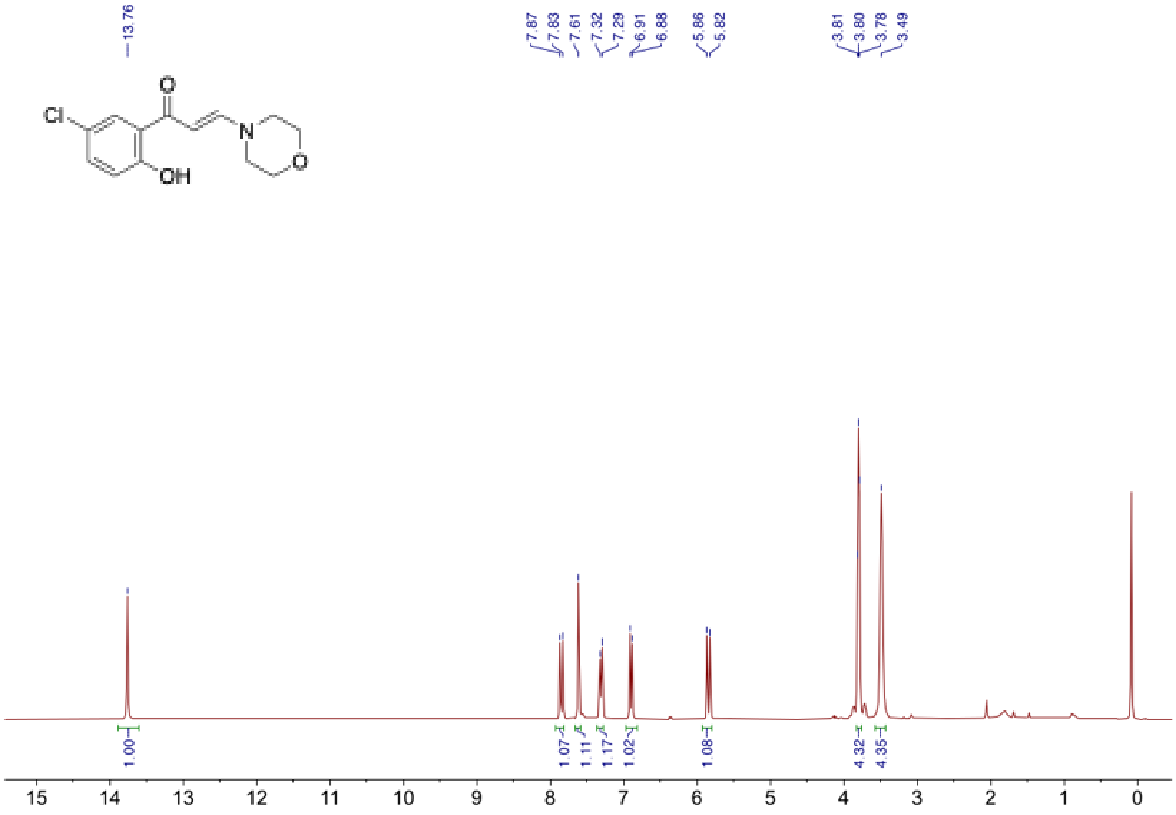

Figure S11: ^{1}H NMR spectra of **9e**.

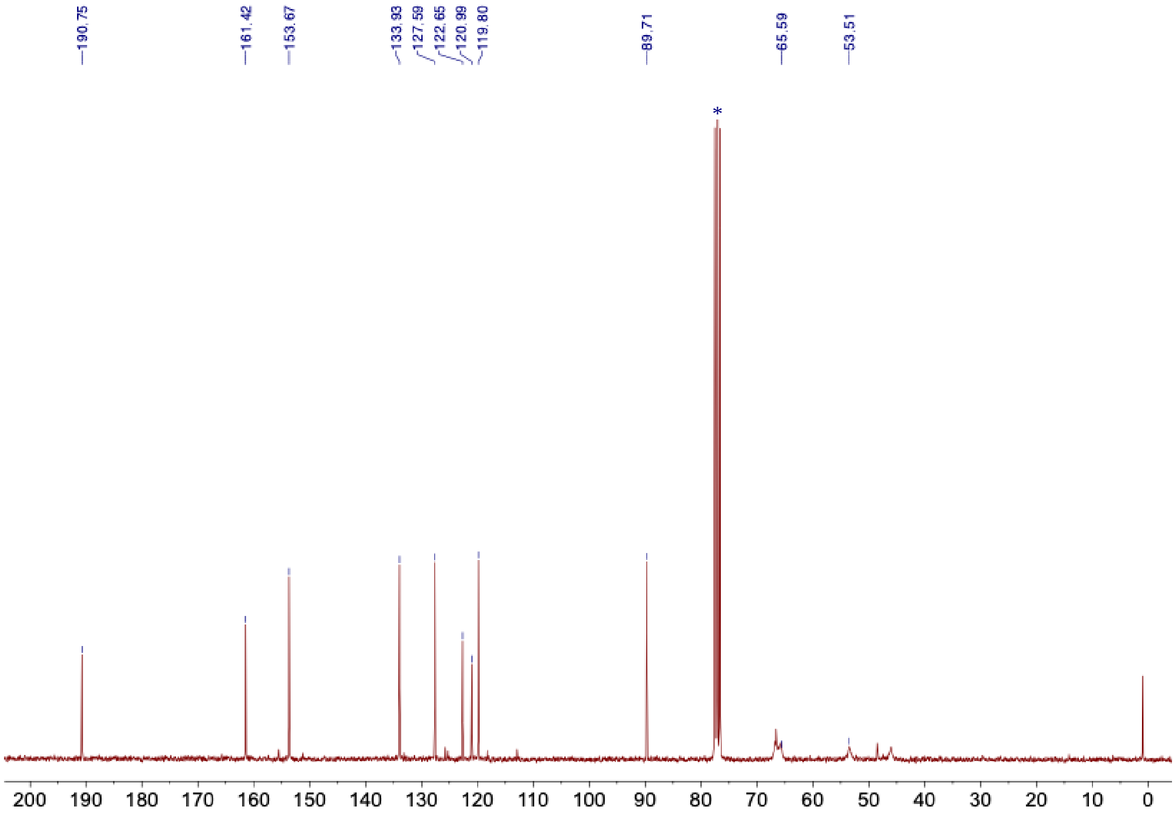

Figure S12: ^{13}C NMR spectra of **9e**.

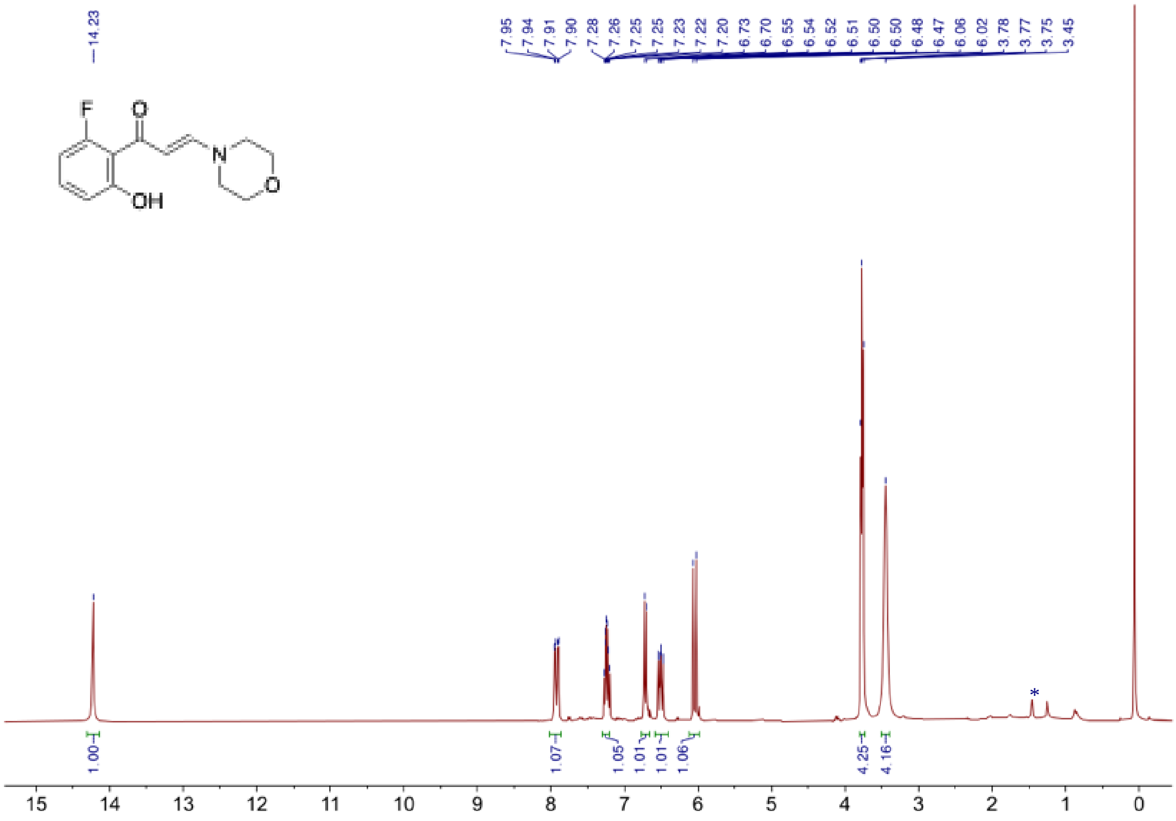

Figure S13: ^{1}H NMR spectra of **9f**.

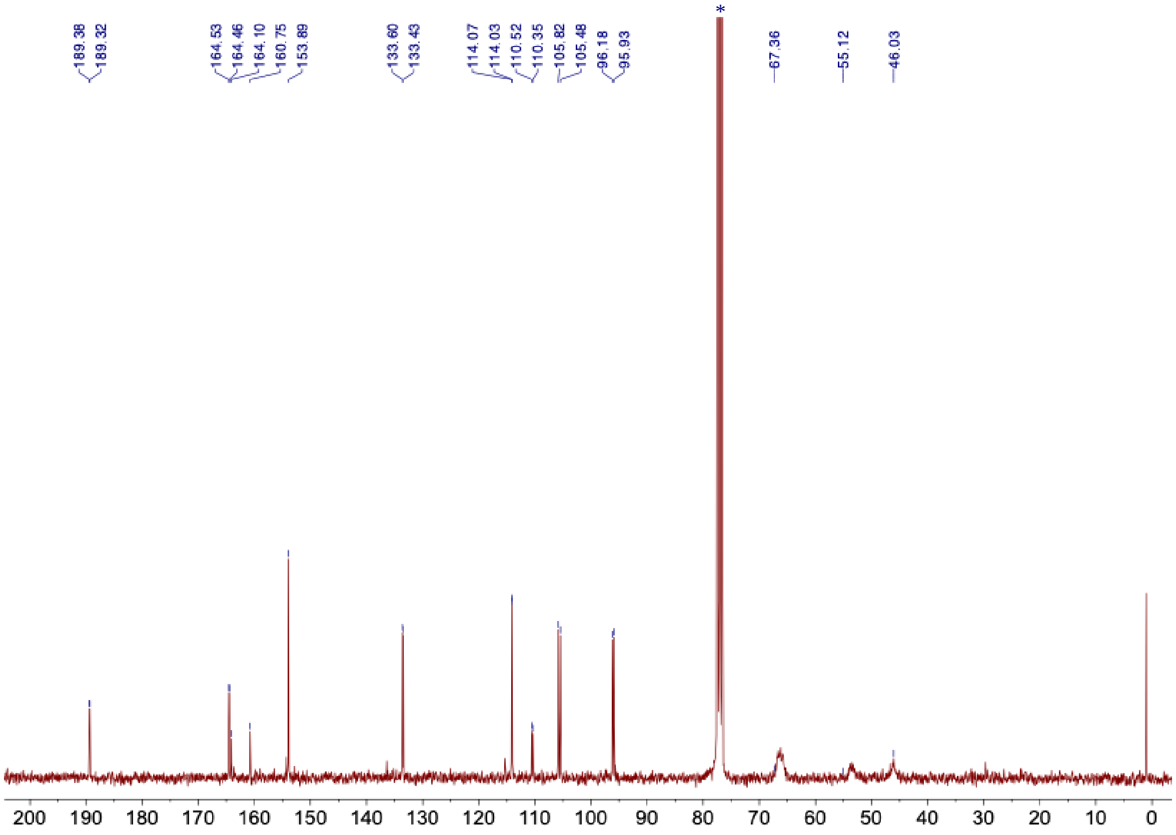

Figure S14: ^{13}C NMR spectra of **9f**.

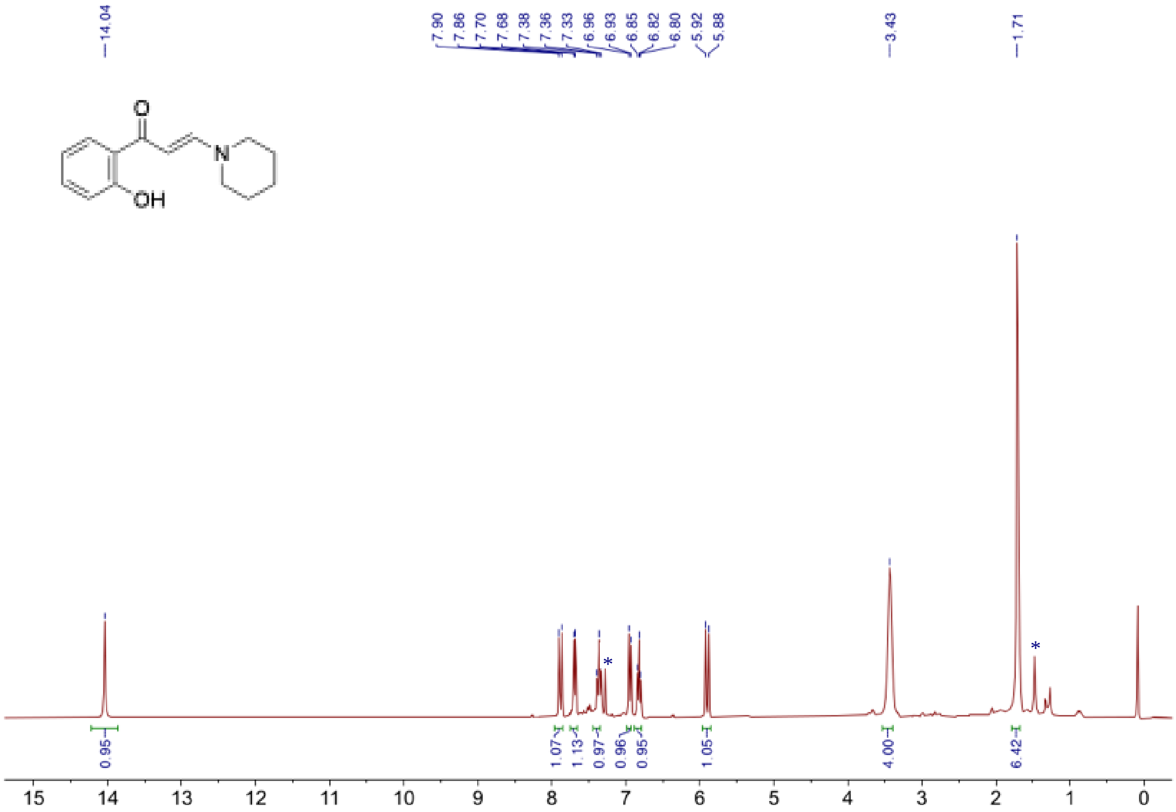

Figure S15: ^{1}H NMR spectra of **9g**.

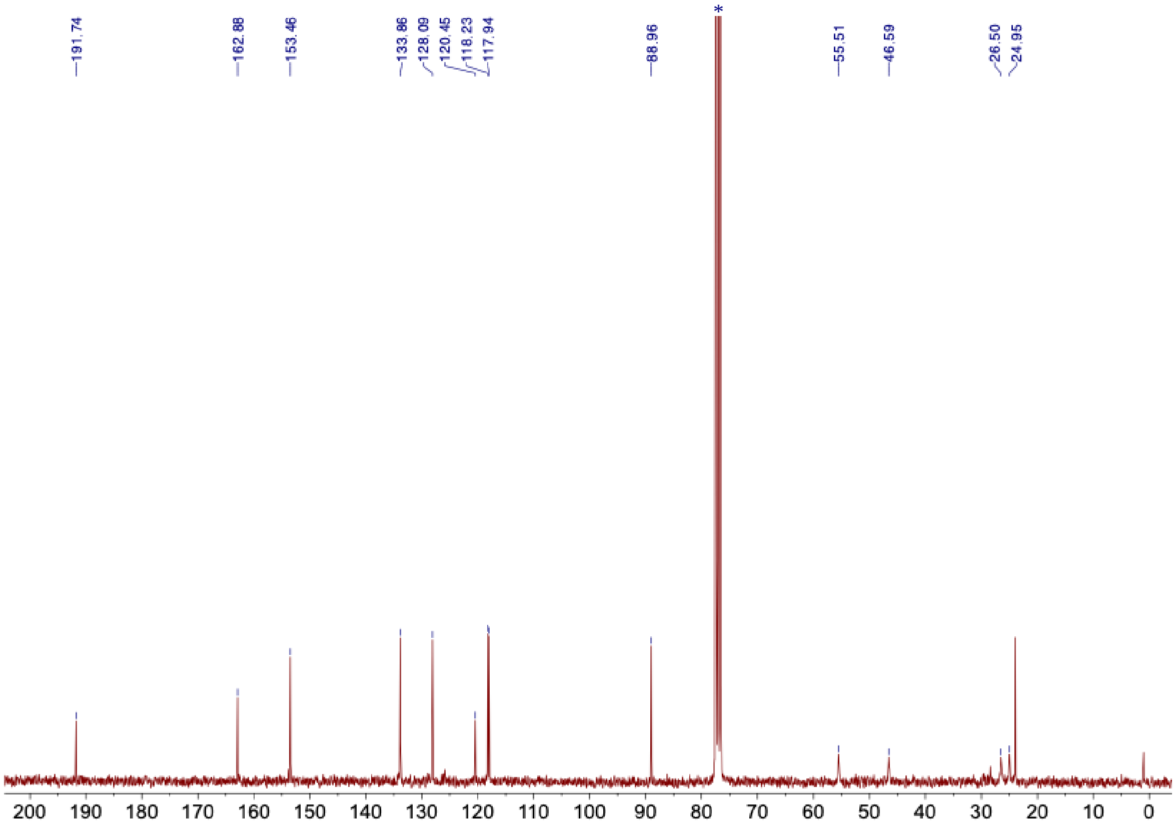

Figure S16: ^{13}C NMR spectra of **9g**.

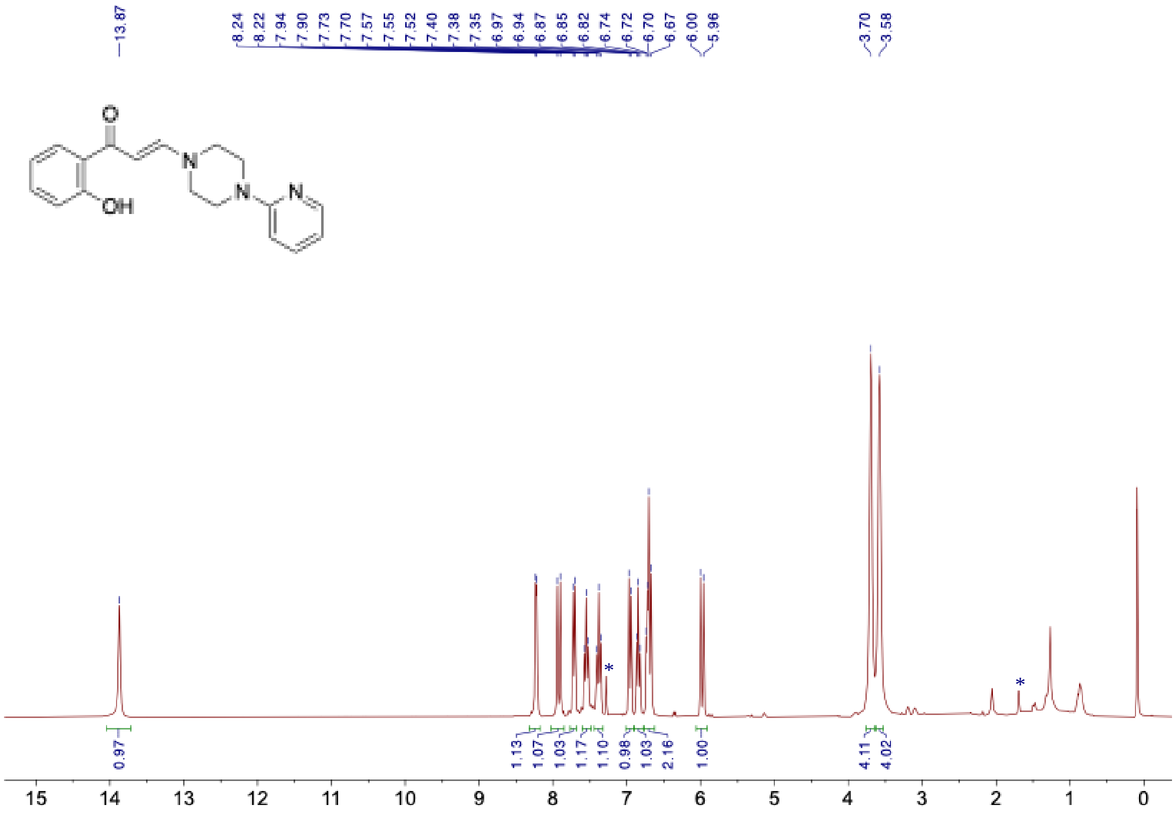

Figure S17: ^{1}H NMR spectra of **9h**.

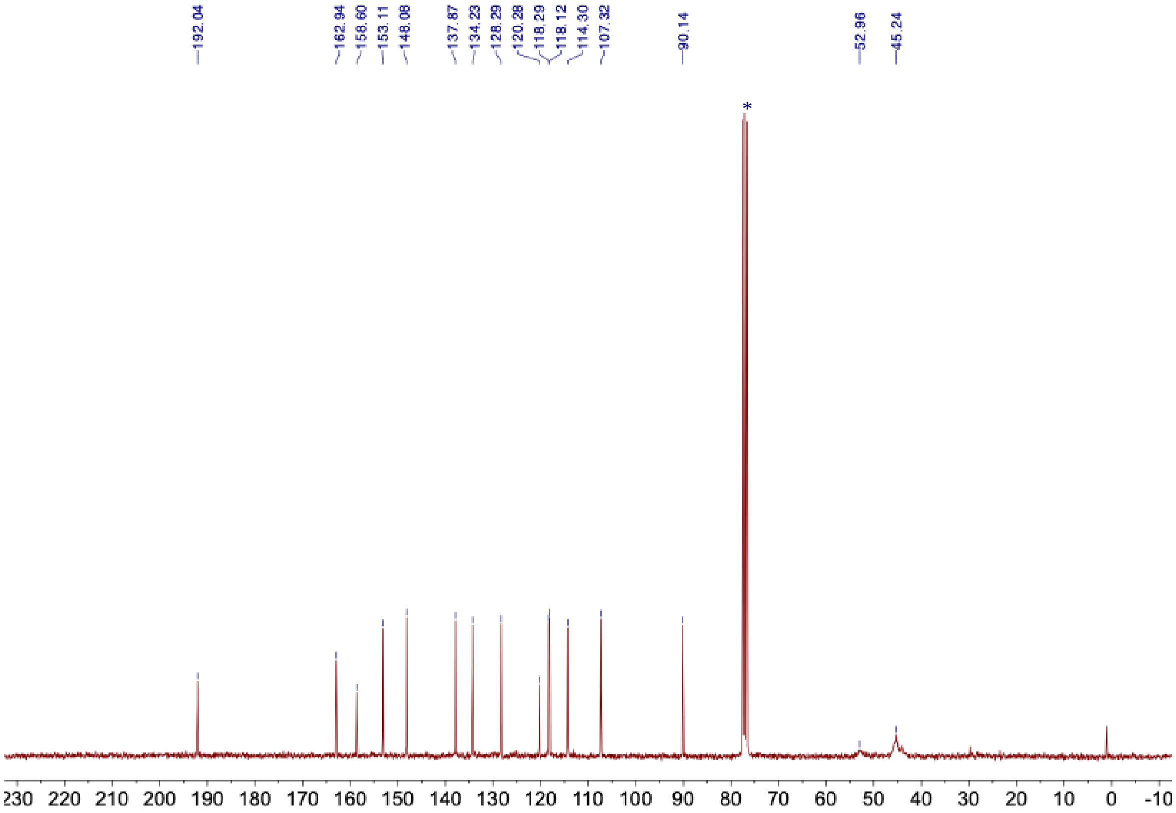

Figure S18: ^{13}C NMR spectra of **9h**.

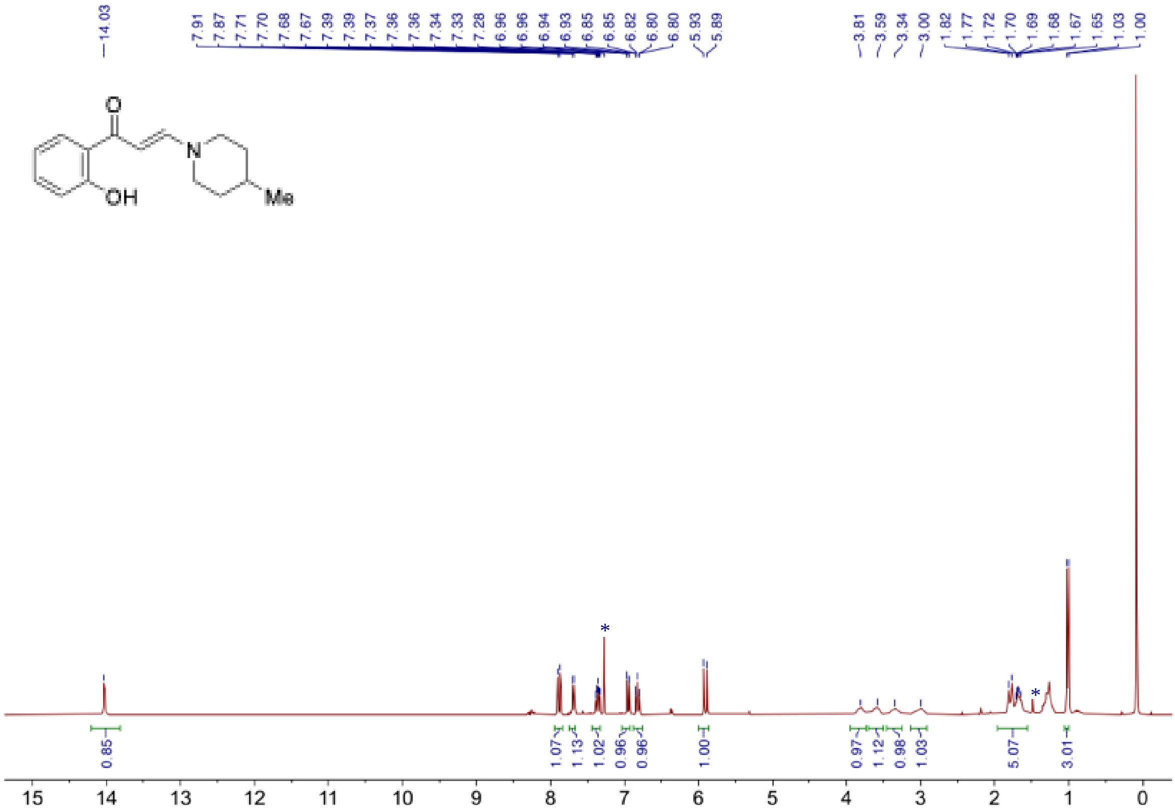

Figure S19: ^{1}H NMR spectra of **9i**.

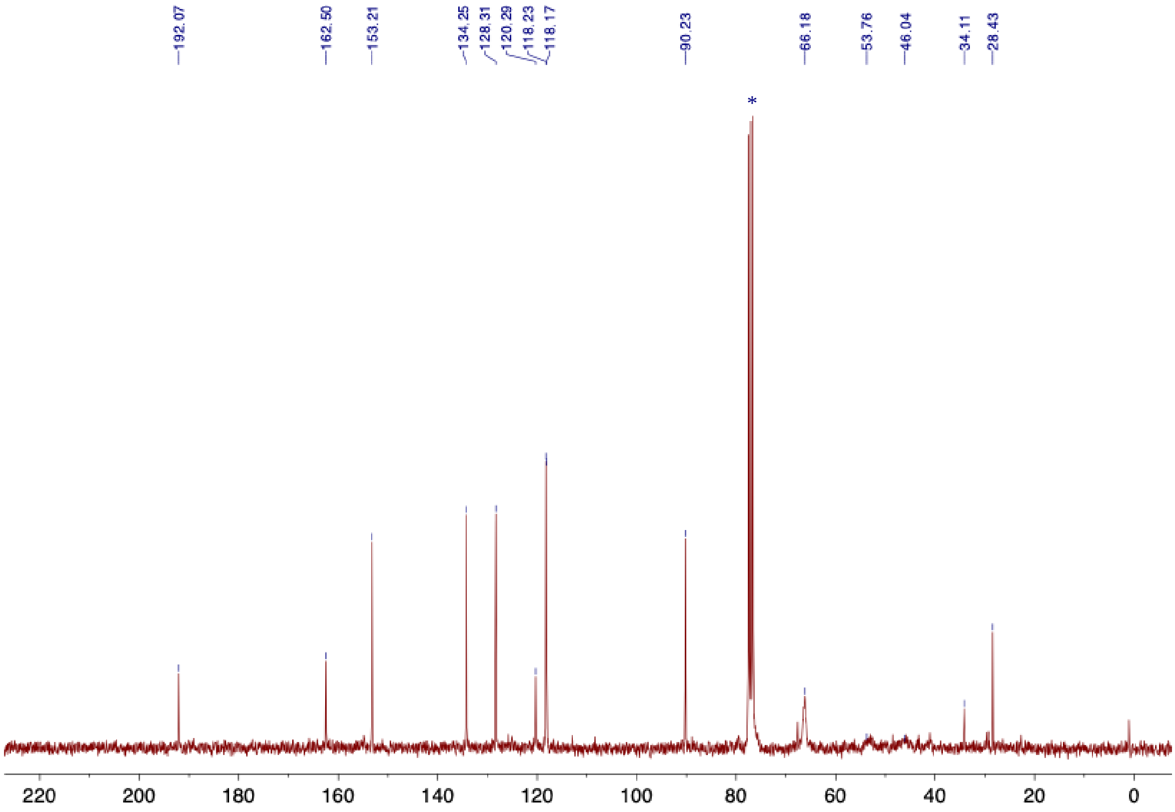

Figure S20: ^{13}C NMR spectra of **9i**.

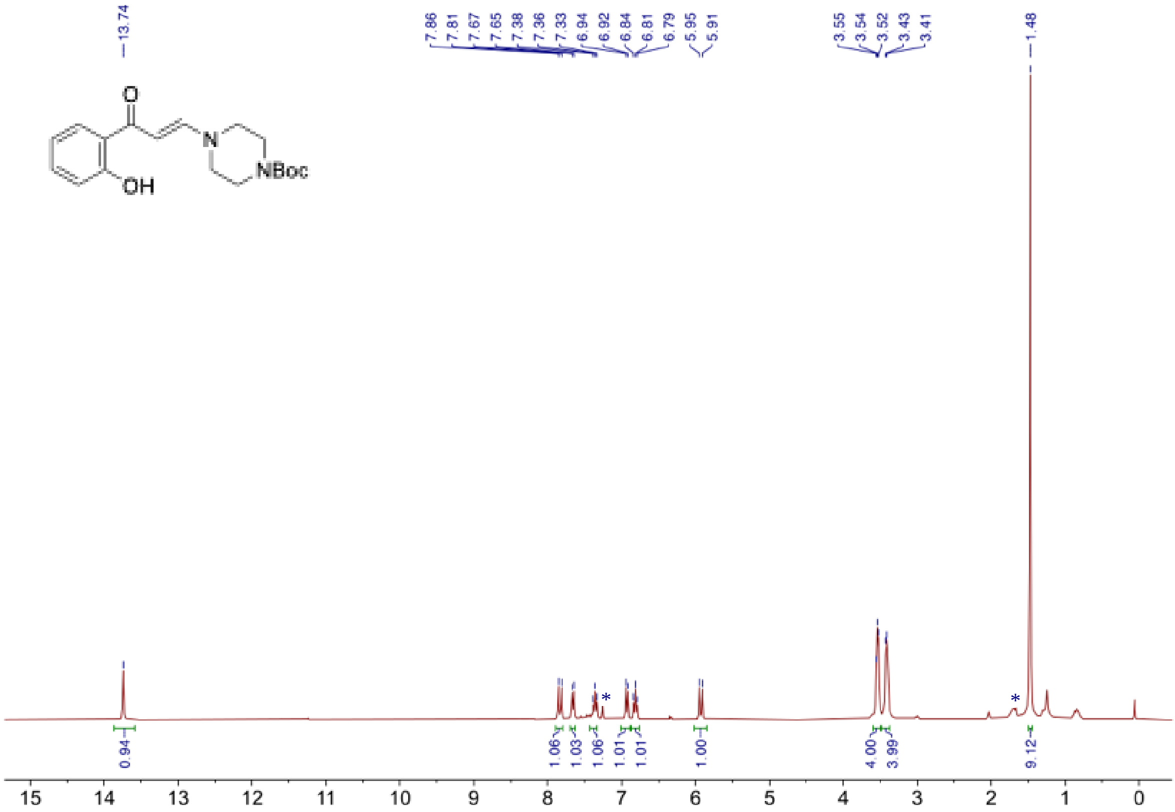

Figure S21: ^{1}H NMR spectra of **9j**.

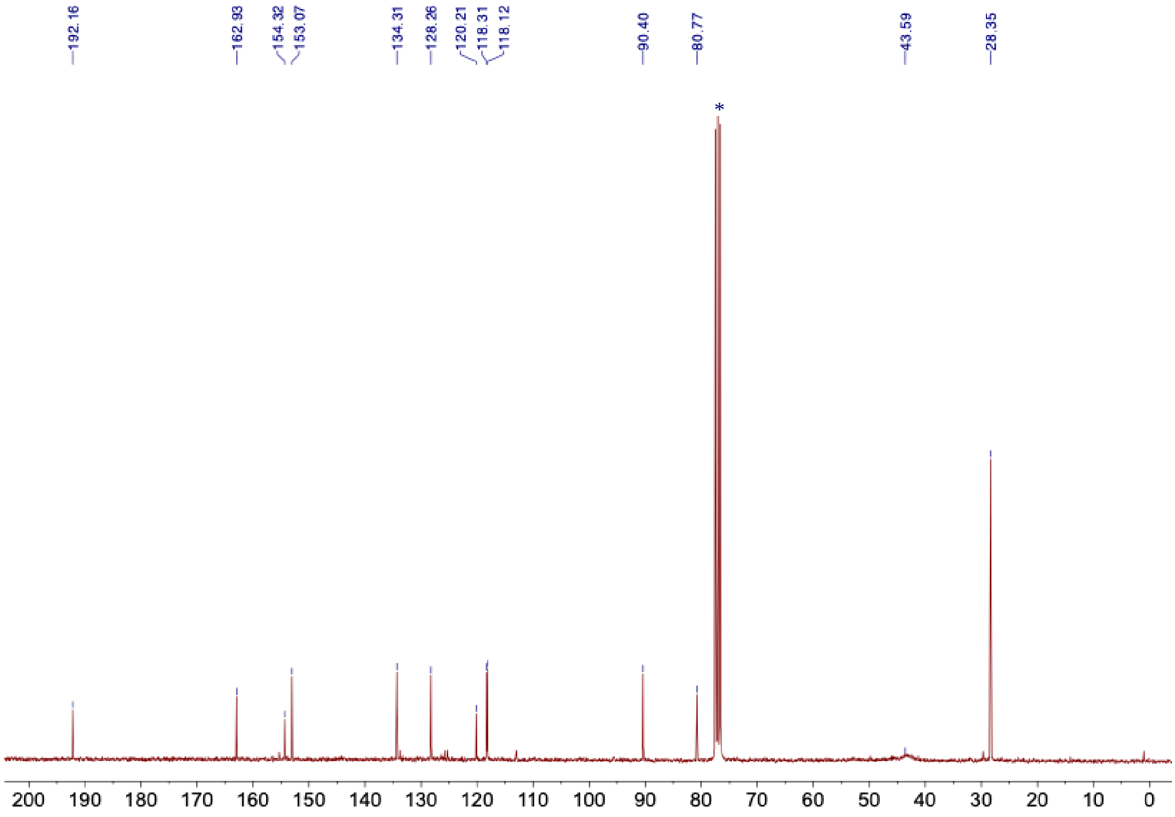

Figure S22: ^{13}C NMR spectra of **9j**.

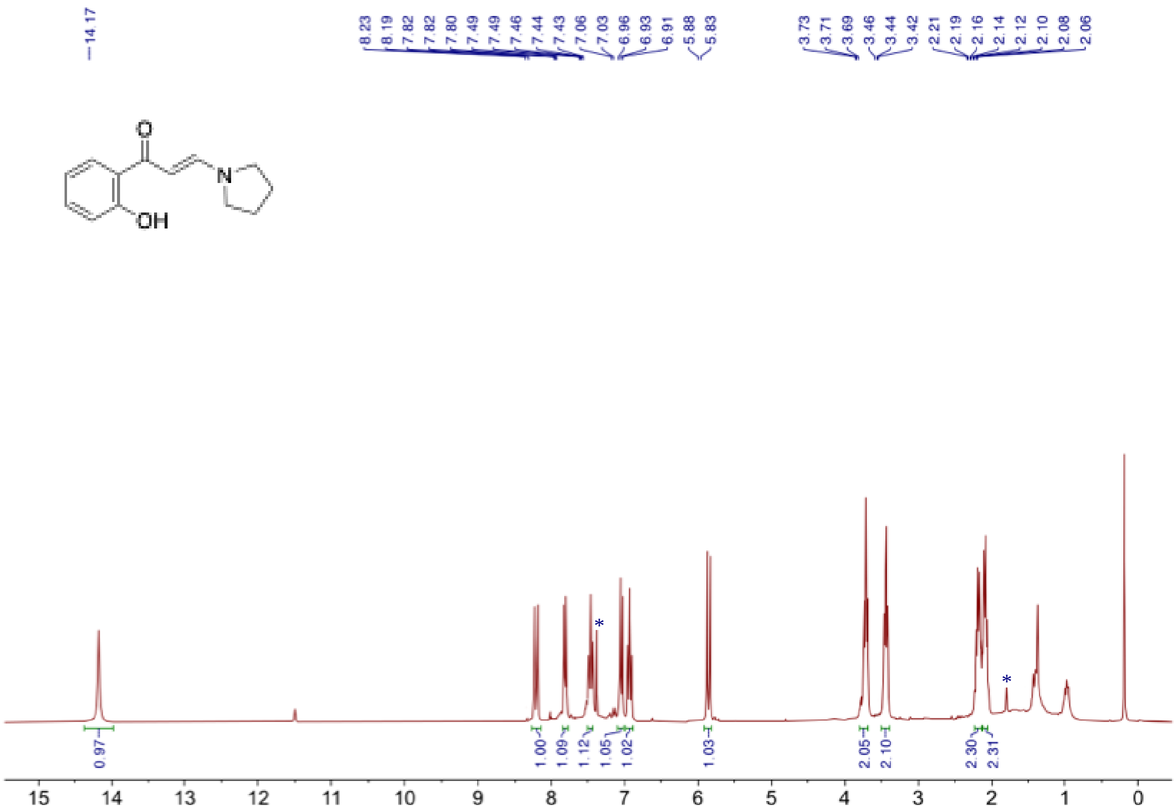

Figure S23: ^{1}H NMR spectra of **9k**.

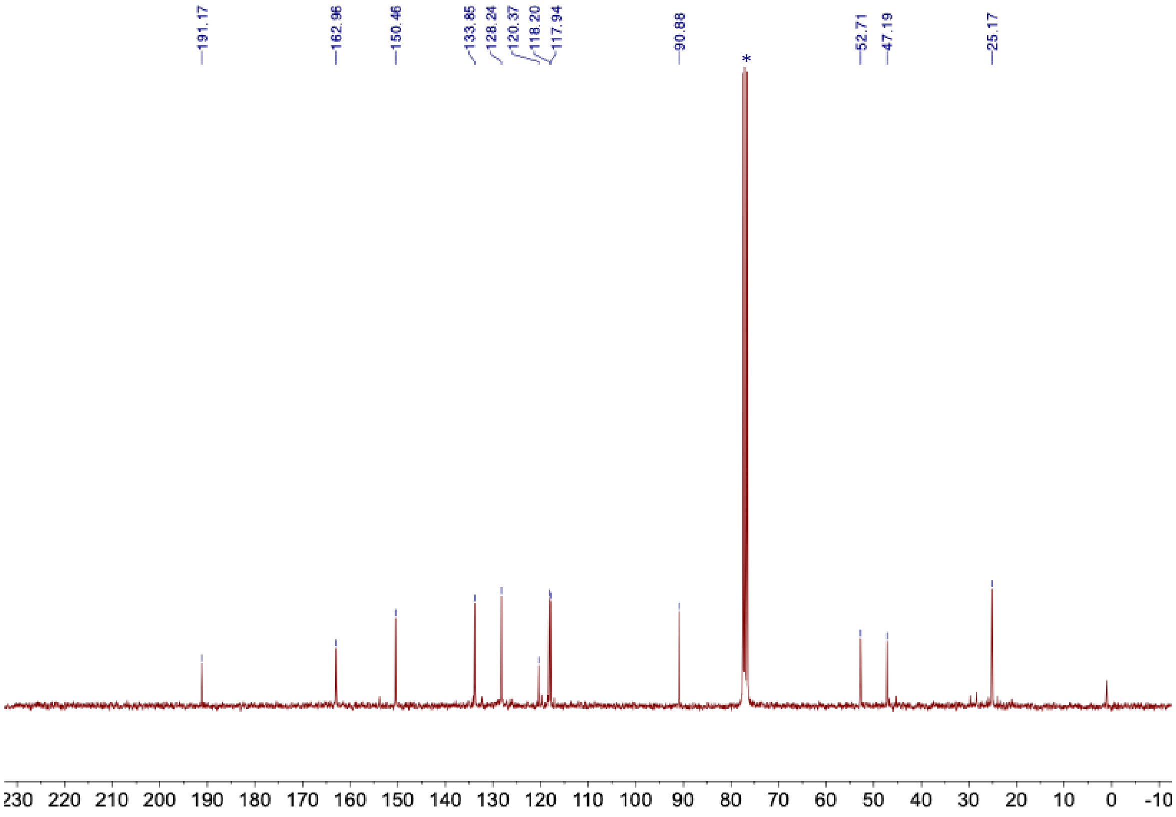

Figure S23: ^{13}C NMR spectra of **9k**.

4. References

[1] Zhao, Y.; Liao, J.; Huang, J.; Xiang, S.; Liu C. *Org. Chem. Front.* **2024**, *11*, 2579–2584.

[2] Pérez-Ramos, P.; Godinho, P. I. C.; Soengas, R. G.; Rodríguez-Solla, H. *RSCAdvances* **2025**, *15*, 15155–15163.

[3] Sosnovskikh, V. Ya.; Irgashev, R. A.; Barabanov, M. A. *Synthesis* **2006**, 2707–2718.

[4] Ghosh, C. K.; Bandyopadhyay, C.; Morin, C. *J. Chem. Soc. Perkin Trans. 1* **1983**, *9*, 1989–1994.

[5] Ghosh, C. K.; Khan, S. *Synthesis* **1981**, 719–721.
